# Metabolic Network Modeling of Microbial Interactions in Natural and Engineered Environmental Systems

**DOI:** 10.3389/fmicb.2016.00673

**Published:** 2016-05-18

**Authors:** Octavio Perez-Garcia, Gavin Lear, Naresh Singhal

**Affiliations:** ^1^Department of Civil and Environmental Engineering, University of AucklandAuckland, New Zealand; ^2^School of Biological Sciences, The University of AucklandAuckland, New Zealand

**Keywords:** environmental biotechnology, systems biology, microbial communities, process engineering, metabolic network, genome-scale metabolic model, flux balance analysis, wastewater treatment

## Abstract

We review approaches to characterize metabolic interactions within microbial communities using Stoichiometric Metabolic Network (SMN) models for applications in environmental and industrial biotechnology. SMN models are computational tools used to evaluate the metabolic engineering potential of various organisms. They have successfully been applied to design and optimize the microbial production of antibiotics, alcohols and amino acids by single strains. To date however, such models have been rarely applied to analyze and control the metabolism of more complex microbial communities. This is largely attributed to the diversity of microbial community functions, metabolisms, and interactions. Here, we firstly review different types of microbial interaction and describe their relevance for natural and engineered environmental processes. Next, we provide a general description of the essential methods of the SMN modeling workflow including the steps of network reconstruction, simulation through Flux Balance Analysis (FBA), experimental data gathering, and model calibration. Then we broadly describe and compare four approaches to model microbial interactions using metabolic networks, i.e., (i) lumped networks, (ii) compartment per guild networks, (iii) bi-level optimization simulations, and (iv) dynamic-SMN methods. These approaches can be used to integrate and analyze diverse microbial physiology, ecology and molecular community data. All of them (except the lumped approach) are suitable for incorporating species abundance data but so far they have been used only to model simple communities of two to eight different species. Interactions based on substrate exchange and competition can be directly modeled using the above approaches. However, interactions based on metabolic feedbacks, such as product inhibition and synthropy require extensions to current models, incorporating gene regulation and compounding accumulation mechanisms. SMN models of microbial interactions can be used to analyze complex “omics” data and to infer and optimize metabolic processes. Thereby, SMN models are suitable to capitalize on advances in high-throughput molecular and metabolic data generation. SMN models are starting to be applied to describe microbial interactions during wastewater treatment, *in-situ* bioremediation, microalgae blooms methanogenic fermentation, and bioplastic production. Despite their current challenges, we envisage that SMN models have future potential for the design and development of novel growth media, biochemical pathways and synthetic microbial associations.

## Introduction

Microbial communities, and the biochemical and ecological interactions occurring in and among them, are ubiquitous in nature. They are vital for human as well as for environmental health, and are manipulated in systems ranging from wastewater treatment plants and agricultural crops to human digestive tracts. Advances in computational tools such as Stoichiometric Metabolic Network (SMN) models and their simulation algorithms [e.g., Flux Balance Analysis (FBA)] are enabling the *in silico* analysis of microbial interactions to enhance desirable metabolic attributes. Community members or metabolic features identified with model predictions can, in theory, be manipulated to control the exchange of metabolic compounds of relevance for environmental protection or industrial applications. Here, we provide an overview of how microbial interactions can be modeled using stoichiometric metabolic networks as well as the main challenges to achieve this. We also describe how such models are starting to be applied to analyze and control natural and engineered environmental systems. Our target audiences are microbiologists, ecologists, and environmental/process engineers who are not currently familiar with metabolic network modeling.

## The big picture

Newer technologies for environmental assessment, waste treatment, and valuable chemical generation are required to achieve equilibrium between socio-economic development and the environment. Growing concerns about the lack of sustainability of past economic growth patterns, and increased awareness of a potential future water and climate crisis, have made clear that the environment and the economy can no longer be considered in isolation from one another. For instance, the global population is expected to increase by 13%, from 7.3 billion in 2015 to 8.3 billion by 2030, (OECD, 2009). This will lead to increased needs for clean water, energy, food, animal feed, fiber for clothing, and housing, therefore putting more strain on our natural environment. As noted in a strategic document by the Organization for Economic Cooperation and Development (OECD, 2011), a return to sustained and sustainable growth will depend upon innovation that delivers a much greener growth model. In this context, environmental and industrial applications of biotechnology, and in particular, applications involving microbial communities, are expected to underpin future innovation. Indeed microbial communities are already developed, used and controlled for the treatment of contaminated environments (land, air, water) and for sustainable manufacturing of valuable chemicals (Kleerebezem and van Loosdrecht, 2007; Miller et al., 2010; Vallero, 2010; Agler et al., 2011; Marshall et al., 2013). SMN modeling, in addition to microbial ecology, fermentation technology, “omics” technologies and process engineering, can assist the development and optimization of many critical microbial processes.

## Environmental processes involving microbial interactions

Members of microbial communities interact with one another by trading metabolites or by exchanging dedicated molecular signals to detect and respond to each other's presence (Brenner et al., 2008). These interactions enable the division of labor whereby the overall output of the community results from the combination of tasks performed by constituent individuals or sub-populations (Teague and Weiss, 2015). Microbial guilds (also called ecological functional groups) are groups of organisms within the community that exploit a class of environmental resource in a similar way. A particular microbial community can be dominated by one or many guilds (Begon et al., 2005). The overall chemical conversions resulting from guilds' metabolic activity (e.g., compound or biomass formation) are termed bioprocesses (Miller et al., 2010). Environmental bioprocesses of human interest are catalyzed by a wide variety of microbial guilds (Table 1) and can be categorized as those involved in major biogeochemical cycles (which are generally applied to remove pollutants from water, soil, and air) and those involved in the production of organic compounds (which are applied for the production of valuable chemicals such as alcohols, methane, or lipids). By interacting with each other, the microbial guilds act as “functional bricks” by which both natural and artificial microbial communities are assembled. Thus, the diversity of environmental bioprocesses is largely defined by the diversity of microbial guilds.

**Table 1:** **Common environmental processes catalyzed by microbial guilds**.

**Catalytic microbial guild**	**Catalyzed environmental process**	**Service/Application**	**Guild's model species**	**References**
Aerobic heterotrophic bacteria	Organic carbon degradation (breakdown of suspended carbon to soluble carbon)	Organic matter removal from wastewater	Bacteroidetes α- and β- proteobacteria, *Acidovorax* spp., *Fermicutes* spp.	Wagner and Loy, 2002; Wagner et al., 2002; Das et al., 2011
	Organic carbon oxidation (soluble carbon to CO_2_)	Organic matter removal from wastewater	Bacteroidetes α- and β- proteobacteria, *Acidovorax* spp., *Fermicutes* spp.	Wagner and Loy, 2002; Wagner et al., 2002; Das et al., 2011
	Proteolysis (organic nitrogen to NH${}_{4}^{+}$)	Global nitrogen cycle, organic matter removal from wastewater	Bacteroidetes α- and β- proteobacteria, *Acidovorax* spp., *Fermicutes* spp.	Wagner and Loy, 2002; Wagner et al., 2002; Das et al., 2011; Schreiber et al., 2012
Heterotrophic denitrifiers	Denitrification (NO${}_{3}^{-}$/NO${}_{2}^{-}$ reduction to N_2_)	Global nitrogen cycle, biological nitrogen removal from wastewater	*Paracoccus denitrifican, Pseudomonas aeruginosa, Acidovorax* spp., α-, and β- Proteobacteria	Ferguson, 1998; Brown, 2010; Kraft et al., 2011; Schreiber et al., 2012
Autotrophic nitrifiers, including both, ammonia oxidizing bacteria (AOB) and nitrite oxidizing bacteria (NOB)	Nitritation (NH${}_{4}^{+}$ oxidation to NO${}_{2}^{-}$)	Global nitrogen cycle, nitrogen removal from wastewater	*Nitrosomonas europaea, Nitrosomonas eutropha, Nitrosospira* spp.	Hooper, 1991; Arp et al., 2002; Chain et al., 2003; Ferguson et al., 2007; Perez-Garcia et al., 2014b
	Nitratation (NO${}_{2}^{-}$ oxidation to NO${}_{3}^{-}$)	Global nitrogen cycle, nitrogen removal from wastewater	*Nitrospira defluvii, Nitrobacter* spp.	Freitag and Bock, 1990; Ferguson et al., 2007; Lücker et al., 2010; Schreiber et al., 2012
	Nitrifier denitrification and hydroxylamine incomplete oxidation (production of NO and N_2_O)	Production and emission green house and ozone depleting gases	*Nitrosomonas europaea, Nitrosomonas eutropha*	Shaw et al., 2006; Yu et al., 2010; Chandran et al., 2011; Schreiber et al., 2012
Anaerobic ammonium oxidizers (ANAMMOX)	Ammonium oxidation to di-nitrogen gas (NH${}_{4}^{+}$ oxidation to N_2_)	Global nitrogen cycle, nitrogen removal from wastewater	*Kuenenia stuttgartiensis, Candidatus* Jettenia asiatica, *Brocardia anammoxidans*	Kuypers et al., 2003; Kuenen, 2008; Hu et al., 2012
Glycogen accumulating organisms (GAOs)	Anaerobic glycogen formation (carbon uptake and storage compound formation without phosphorus release)	Phosphorus removal from wastewater	*Micropruina glycogenica, Tetrasphaera* spp., *Amaricoccus* spp.	Seviour et al., 2003; de-Bashan and Bashan, 2004; Martín et al., 2006; Wilmes et al., 2008
Phosphate accumulating organisms (PAOs)	Anaerobic phosphorus release (hydrolysis of intracellular polyphosphates for carbon uptake and storage compound formation)	Phosphorus removal from wastewater	*Acinetobacter* spp., *Microlunatus phosphovorus, Clostridium* spp.*, Candidatus* Accumulibacter phosphatis	Seviour et al., 2003; de-Bashan and Bashan, 2004; Martín et al., 2006; Wilmes et al., 2008
	Aerobic phosphorus uptake (storage compound degradation accompanied by soluble phosphorus uptake)	Phosphorus removal from wastewater	*Acinetobacter* spp., *Microlunatus phosphovorus, Candidatus* Accumulibacter phosphatis	Seviour et al., 2003; de-Bashan and Bashan, 2004; Martín et al., 2006; Wilmes et al., 2008
Polyhydroxyalkanoates (PHA) accumulating bacteria	Anaerobic formation of carbon storage compounds in form of polymers of the PHA family	Polyhydroxybutyrate (PHB) base bioplastic production	*Pseudomonas oleovorans, Alcaligenes eutrophus, Azotobacter vinelandii, Alcaligenes latus*	Batstone et al., 2003; Patnaik, 2005; Dias et al., 2008
Hydrogen producing acetogenic bacteria/archea	Fermentation of higher organic acids to produce acetate, H_2,_ and CO_2_	Hydrogen and methane production	*Clostridium* spp., *Syntrophomonadaceae* spp., Bacteriodetes	Hatamoto et al., 2007; Rittmann et al., 2008; Khanal, 2009a,b
Autotrophic homoacetogenic bacteria	Syngas fermentation (use of hydrogen carbon monoxide and dioxide as carbon and energy source)	Ethanol, butanol, methane and small chain fatty acid production	*Clostridium ljungdahlii*	Khanal, 2009b; Abubackar et al., 2011
Heterotrophic homoacetogenic bacteria	Fermentation of higher organic acids and alcohols to produce acetate and CO_2_	Methane production	Streptococcaceae and Enterobacteriaceae families, *Clostridium aceticum, Acetobacterium woodii*, and *Bacteroidetes* spp., *Clostridium* spp., *Lactobacillus* spp.	Hatamoto et al., 2007; Rittmann et al., 2008; Khanal, 2009a,b
Anaerobic methanogenic archea	Acetotrophic conversion of acetate to methane	Methane production	*Methanosarcina* spp. and *Methanosaeta* spp.	(Hatamoto et al., 2007; Rittmann et al., 2008; Khanal, 2009a,b)
	Hydrogenotrophic conversion of carbon dioxide to methane	Methane production	*Methanosarcina* spp	Hatamoto et al., 2007; Rittmann et al., 2008; Khanal, 2009a,b
Photo-autotrophs (Microalgae/Cyanobacteria)	Nutrient assimilation (soluble N & P assimilation to organic molecules)	Eutrophication of water bodies, nutrient removal from wastewater	*Clamydomonas reinhardtii, Chlorella vulgaris, Spirulina platensi, Microcystis aeruginosa, Anabaena* spp., *Oscilatoria* spp., *Nostoc* spp.	de-Bashan and Bashan, 2004, 2010; Perez-Garcia et al., 2010
	Autotrophic CO_2_ fixation (CO_2_ fixation to biomass)	Global carbon cycle, biomass formation, CO_2_ sequestration	*Clamydomonas reinhardtii, Chlorella* spp.*, Spirulina platensi, Microcystis aeruginosa, Scenedesmus obliquus, Nanochloropsis* spp.	Das et al., 2011; Cheirsilp and Torpee, 2012; Girard et al., 2014; Wu et al., 2015
	Autotrophic and heterotrophic lipid, starch and pigments production	Biofuels and valuable chemical production	*Chlorella vulgaris, Chlorella prototecoides*	de-Bashan et al., 2002; Perez-Garcia et al., 2011; Choix et al., 2012a,b; Perez-Garcia and Bashan, 2015
	Production of nitrous and nitrous oxides	Production and emission green house and ozone depleting gases	*Chlorella vulgaris*	Guieysse et al., 2013; Alcántara et al., 2015
	Synthesis of exo-polymers	Bio-absorption of organic compounds and pollutants	*Clamydomonas reinhardtii, Chlorella vulgaris, Spirulina platensis*.	Markou and Georgakakis, 2011; Subashchandrabose et al., 2013
Cyanobacteria	Production and realize of secondary metabolites and toxic organic compounds (microcystin, nodularin, cylindrospermopsin, among others)	Self-population an grazer organism control	*Microcystis aeruginosa, Anabaena* spp., *Oscilatoria* spp., *Nostoc* spp.	Welker and Von Döhren, 2006; Yadav et al., 2009; Kaplan et al., 2012; Dittmann et al., 2013; Neilan et al., 2013
Dissimilatory metal-reducing bacteria.	Anaerobic Fe^3+^ reduction to Fe^2+^ (reduction of insoluble iron to soluble form)	Global iron cycle, bioremediation of metallic pollutants in soil and groundwater	*Geobacter metallireducens, Geobacter sulfurreducens, Albidoferax ferrireducens, Shewanella putrefaciens*	Lovley and Coates, 1997; Malik, 2004; Gadd, 2010; Melton et al., 2014
	Anaerobic Mn^4+^ reduction to Mn^2+^ (reduction of insoluble iron to soluble form)	Global iron cycle, bioremediation of metallic pollutants in soil and groundwater	*Geobacter metallireducens, Geobacter sulfurreducens, Albidoferax ferrireducens, Shewanella putrefaciens*	Lovley and Coates, 1997; Malik, 2004; Gadd, 2010; Melton et al., 2014
	Anaerobic As^5+^ reduction to As^3+^ (reduction of insoluble arsenic to soluble)	Bioremediation of metallic pollutants in soil	*Geospirillum arsenophilus, Geospirillum barnseii, Chrysiogenes arsenatis, Sulfurospirillum* strain NP4	Lovley and Coates, 1997; Malik, 2004; Lear et al., 2007; Gadd, 2010
	Aerobic Hg^2+^ reduction to Hg^0^ (reduction of soluble mercury to volatile form)	Bioremediation of metallic pollutants in soil and water	*Pseudomonas* spp.	Lovley and Coates, 1997
	Anaerobic U^6+^ reduction to U^4+^ (reduction of soluble uranium to insoluble form)	Soil bioremediation of radioactive pollutants	*Thiobacillus thiooxidan, Rhodoferax ferrireducens, Geobacter sulfurreducens, Shewanella putrefaciens, Desulfotomaculum reducens*	Lovley and Coates, 1997; Malik, 2004; Gadd, 2010
	Anaerobic Tc^4+^ reduction to Tc^7+^ (reduction of soluble technecium to poorly soluble form)	Soil bioremediation of radioactive pollutants	*Geobacter* spp.	Lear et al., 2010
	Anaerobic and aerobic Cr^6+^ reduction to Cr^3+^ (reduction of soluble chromium to insoluble form)	Bioremediation of metallic pollutants in soil and water	*Pseudomonas* spp., *Achromobacter Eurydice, Desulfovibrio vulgaris, Bacillus* spp., *Desulfotomaculum reducens*	Wang and Shen, 1995; Lovley and Coates, 1997; Malik, 2004; Gadd, 2010
Heavy metal resistant microbes	Heavy metal (Cu, Zn, Ni, Cd, Pb, Hg) immobilization by biosorption, bioaccumulation, biochelation	Bioremediation of metallic pollutants in soil and water	*Alcaligenes eutrophus, Alcaligenes xylosoxidans, Stenotrophomonas* sp., *Ralstonia eutropha, Staphylococcus* sp., *Pseudomonas syringae*	Lovley and Coates, 1997; Diels et al., 1999; Malik, 2004; Gadd, 2010; Edwards and Kjellerup, 2013
Dissimilatory sulfate reducing bacteria	Anaerobic SO${}_{4}^{2-}$ reduction to H_2_S (reduction of soluble and insoluble sulfur to volatile form)	Global sulfur cycle, treatment of sulfur and sulfate contaminated groundwater and industrial wastewater	*Desulfovibrio* spp., *Thermodesulfovibrio yellowstonii, Archaeoglobus* spp., *Desulfatibacillum* spp. *Desulfothermus* spp., *Desulfotomaculum reducens*	Lovley and Coates, 1997; Malik, 2004; Gadd, 2010; Pereira et al., 2011; Hao et al., 2014
Sulfur oxidizing bacteria	Chemiolitotrophic H_2_S, S^0^ oxidation to SO${}_{4}^{2-}$ (reduction of soluble and insoluble sulfur to volatile form)	Global sulfur cycle, bioremediation of sulfur pollutants in water	*Beggiatoa* spp., *Thiobacillus novellus, Sulfolobus* spp., Purple and green sulfur-oxidizing bacteria	Lovley and Coates, 1997; Kappler et al., 2000; Malik, 2004; Gadd, 2010; Pokorna and Zabranska, 2015
Iron oxidizing bacteria	Chemiolitotrophic Fe^2+^ oxidation to Fe^3+^ (oxidation of soluble iron to insoluble form)	Global iron cycle, bioremediation of metallic pollutants in water	*Leptospirillum ferrooxidans, Acidithiobacillus ferrooxidans, Sulfobacillus thermosulfidooxidans*	Lovley and Coates, 1997; Malik, 2004; Gadd, 2010
Ectomycorrhizal fungi	Filamentous (hyphae) extension of plant root systems (do not penetrate plant root cells)	Enhance plant acquisition of nitrogen, minerals and water	*Russula xerampelina, Amanita francheti, Suillus bovinus*	Gardes and Bruns, 1996; Chalot and Brun, 1998; Reid and Greene, 2012
Arbuscular mycorrhizae fungi	Filamentous (hyphae) extension of plant root systems (penetrate plant root cells)	Enhance plant acquisition of nutrients, minerals and water	*Rhizophagus Irregularis, Piriformospora indica*	Reid and Greene, 2012
Endophytic fungi	Fungi-plant symbiotic production of bioactive compounds	Pathogen and predator resistance	Clavicipitaceae family	Reid and Greene, 2012
Lignocellulosic fungi	Lignin degradation to soluble carbohydrates mediated by peroxidases and laccase	Global carbon cycle, lignocellulosic biomass degradation, biofuel production, bio-refining of valuable chemicals	*Phanerochaete chrysosporium, Pleurotus* spp.*, Trametes versicolor, Phanerochaete chrysosporium*	Bugg et al., 2011; Harms et al., 2011
	Organic pollutant degradation to harmless compounds mediated by peroxidase, laccase and cytochromes	Organic pollutant degradation, bioremediation	*Gloeophyllum* spp., *Trabeum* spp., *Gliocladium virens, Trametes versicolor, Phanerochaete chrysosporium, Candida* spp.	Keller et al., 2005; Bugg et al., 2011; Harms et al., 2011; Lah et al., 2011; Margot et al., 2013
Recalcitrant pollutant degrading bacteria	Organic pollutant degradation to harmless compounds mediated by peroxidase, laccase, and cytochromes	Organic pollutant degradation (pesticides, pharmaceuticals, agrochemicals, industrial waste chemicals, oil, and petrochemicals)	*Pseudomonas* spp., *Streptomyces* spp., *Desulfovibrio* spp., *Brevundimonas diminuta*,	Díaz, 2004; Head et al., 2006; Singh, 2009; Guazzaroni and Ferrer, 2011; Nikel et al., 2014
Plant growth promoting bacteria (PGPB)	Diazotrophic nitrogen fixation (di-nitrogen gas conversion to ammonia, which is available for plant assimilation)	Global nitrogen cycle, increase biomass production yields of plants or microalgae	*Azospirillum brasilense, Azospirillum lipoferum, Bacillus pumilus, Azoarcus* sp., *Rhizobium leguminosarum*	Hartmann and Bashan, 2009; Hernandez et al., 2009; Reid and Greene, 2012
Plant and microalgae promoting bacteria	Phytohormone production (indole-3-acetic acid and gibberellin production)	Increase of starch formation, and ammonium and phosphate uptake by microalgae	*Azospirillum brasilense, Bacillus pumilus*	de-Bashan et al., 2002, 2005, 2011; Choix et al., 2012a, 2014; Meza et al., 2015a,b

*Communities' microbial guilds can be modeled using metabolic networks by rendering genomic data of model species enlisted in the fourth column*.

### Definition of microbial interactions

Guilds or species interactions in microbial communities can be either metabolism-based or be driven by ecological traits. Several good reviews have summarized the study of ecological interactions among microbes in synthetic as well as in natural microbial communities (Faust and Raes, 2012; Mitri and Richard Foster, 2013). Here, we emphasize the role of metabolism in driving species interactions and *vice versa*, since metabolism-based views allow the creation of both theoretical mechanistic models and experimental manipulation.

**Figure 1:**
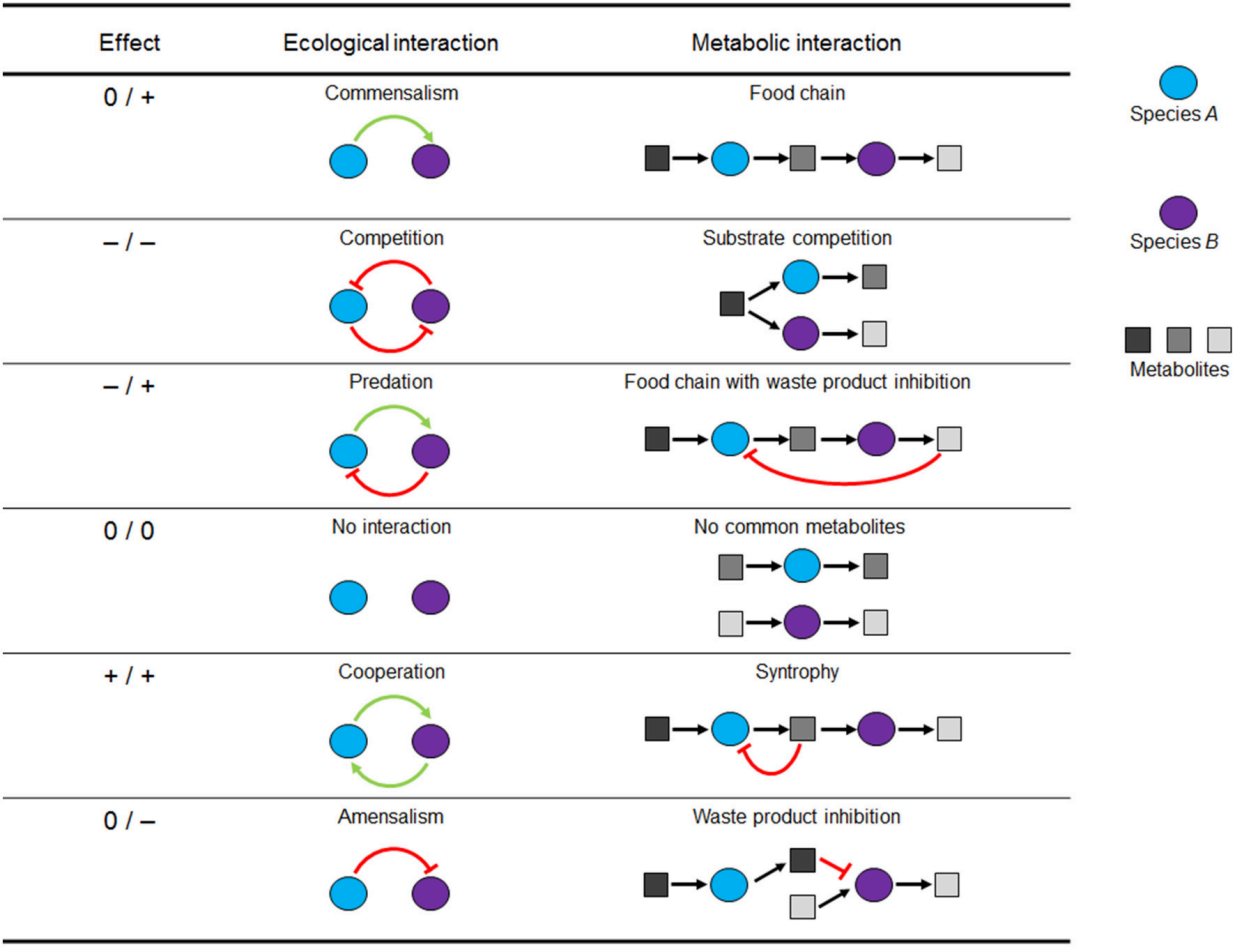
**Pairwise microbial interactions in environmental processes**. For each interaction partner, there are three possible outcomes: positive (+), negative (–), or neutral (0). Metabolic but not ecological interactions can be modeled using metabolic networks. Figure adapted from Großkopf and Soyer (2014).

The net effect of the metabolic interaction between species/guild A on a second species/guild B can be positive (+), negative (–) or neutral (0, no impact on the species involved) (Faust and Raes, 2012; Großkopf and Soyer, 2014). The possible combinations of win, lose, or neutral outcomes for two interaction partners allow the classification of nine interaction types. However, if interaction directionality is neglected (i.e., +/– is considered the same as –/+), there are six basal interaction patterns. Figure 1 depicts these interaction patterns by ecological and a corresponding metabolic representation (i.e., the communication between species via their metabolic products). Since, the combinatorial explosion of possible interaction states quickly reaches large numbers with only a few species, the challenge is to find key interactions that are over-represented in nature or that can have significant percolating effects at the community level (e.g., stabilizing or de-stabilizing interactions) (Großkopf and Soyer, 2014). Indeed, microbial interaction networks, like human social networks, generally imply the presence of many taxa with only a few links and a few highly connected (hub) taxa (Faust and Raes, 2012; Großkopf and Soyer, 2014). The definitions of these microbial interactions are important, allowing them to be formally described for use in SMN modeling frameworks.

### Use of microbial interactions in engineered processes

Engineered processes relying on microbial communities have been around for nearly a century. Microbial interactions are intentionally stabilized by selecting the source of the microbial inoculum and by controlling environmental conditions to promote the selection of favorable microbial taxa and processes (Rodríguez et al., 2006; Kleerebezem and van Loosdrecht, 2007). The use and stabilization of microbial communities for bioprocessing can have clear advantages over the use of traditional pure cultures (Rodríguez et al., 2006; Kleerebezem and van Loosdrecht, 2007; de-Bashan et al., 2011; Marshall et al., 2013). Such advantages are: (i) no sterilization requirement, reducing operational costs; (ii) the capacity to use cheap, mixed, or complex substrates; (iii) greater adaptive capacity (a larger pool of genes allows different processes to be performed depending on the environmental conditions); (iv) increased process robustness; (v) performance of complicated tasks (division of labor and metabolic modularity allow several processes to occur in a single culture); and (vi) that controlling interactions allows process regulation. The above advantages confirm cultures of microbial communities (a.k.a. mixed microbial cultures) are an attractive platform for the discovery and development of new bioprocesses. For instance, the use of open mixed microbial cultures (MMC) and less-pure or waste materials as substrate can substantially decrease the cost of polyhydroxyalkanoates (PHA) or microalgae based products and therefore increase their market potential and positive environmental outcomes (Dias et al., 2005; Rodríguez et al., 2006; Pardelha et al., 2012; Perez-Garcia and Bashan, 2015). Anaerobic digestion is a classic example of a process that combines the objectives of elimination of organic compounds from a waste stream with the generation of a valuable product in the form of methane-containing biogas (Kleerebezem and van Loosdrecht, 2007). Bioprocesses based on MMC exhibit robustness and reproducibility, which is highly desirable in industrial applications (Allison and Martiny, 2008; Werner et al., 2011). Additionally, The physicochemical properties of bioreactor feed may select the most efficient and effective microbial catalysts and even lead to the evolution of more stable and productive microbial communities (Marshall et al., 2013). For instance, biological wastewater treatment by activated sludge and bioreactors for PHA production can operate continuously for years.

### Limitations of processes based on microbial interactions

Despite the above-mentioned advantages, environmental processes based on microbial communities are currently not widely applied at industrial scale—except for wastewater treatment and anaerobic biodigesters—as this technology still presents significant difficulties. The products formed by microbial communities vary in amount and composition and can have low market value (Kleerebezem and van Loosdrecht, 2007; Agler et al., 2011). Control of the optimum balance among the microorganisms is not straightforward and requires a better understanding of microbial community behavior (Agler et al., 2011). In some MMC processes, the observed yields are much lower than the ones observed from pure cultures or expected from the theoretical process reaction stoichiometry. For example, in anaerobic bio-hydrogen production from carbohydrates, the measured hydrogen production per mole of glucose is much lower (two moles) than the theoretical four mol-H/mol-Glucose yield expected from the bioprocess reaction stoichiometry (Li and Fang, 2007). Another disadvantage is that metabolic routes for waste degradation or product formation can be undefined, therefore complicating the implementation of operation strategies (Rodríguez et al., 2006; Li and Fang, 2007). Therefore, even though mixed microbial cultures are attractive for bioprocessing, the above negative aspects challenge their wider application. In this context, mathematical modeling of metabolic conversions, and interactions can assist to overcome these limitations.

## Stoichiometric metabolic network (SMN) models

### Bioprocess modeling and metabolic modeling

Natural and engineered environmental processes are complex systems that depend on external chemical and physical factors. The problem of complexity can be addressed with mathematical models that enable simulation (prediction) of process behavior. So that it is possible to estimate the impact that changing independent variables (e.g., biomass retention time, key nutrient concentrations, pH or temperature) will have on the service or product of interest (Makinia, 2010). Mathematical modeling of bioprocesses is a common practice in environmental engineering. For instance, the International Water Association's Activated Sludge Models (ASM) are a family of bioprocess models widely used by researchers and wastewater treatment facility operators (Kaelin et al., 2009; Makinia, 2010). The main applications of ASM models are, according to van Loosdrecht et al. (2008): to gain insight into process performance and to evaluate possible scenarios for process and plant upgrading. Given such applications, mathematical modeling is a powerful tool to address the complexity and poor reproducibility of environmental processes.

**Figure 2:**
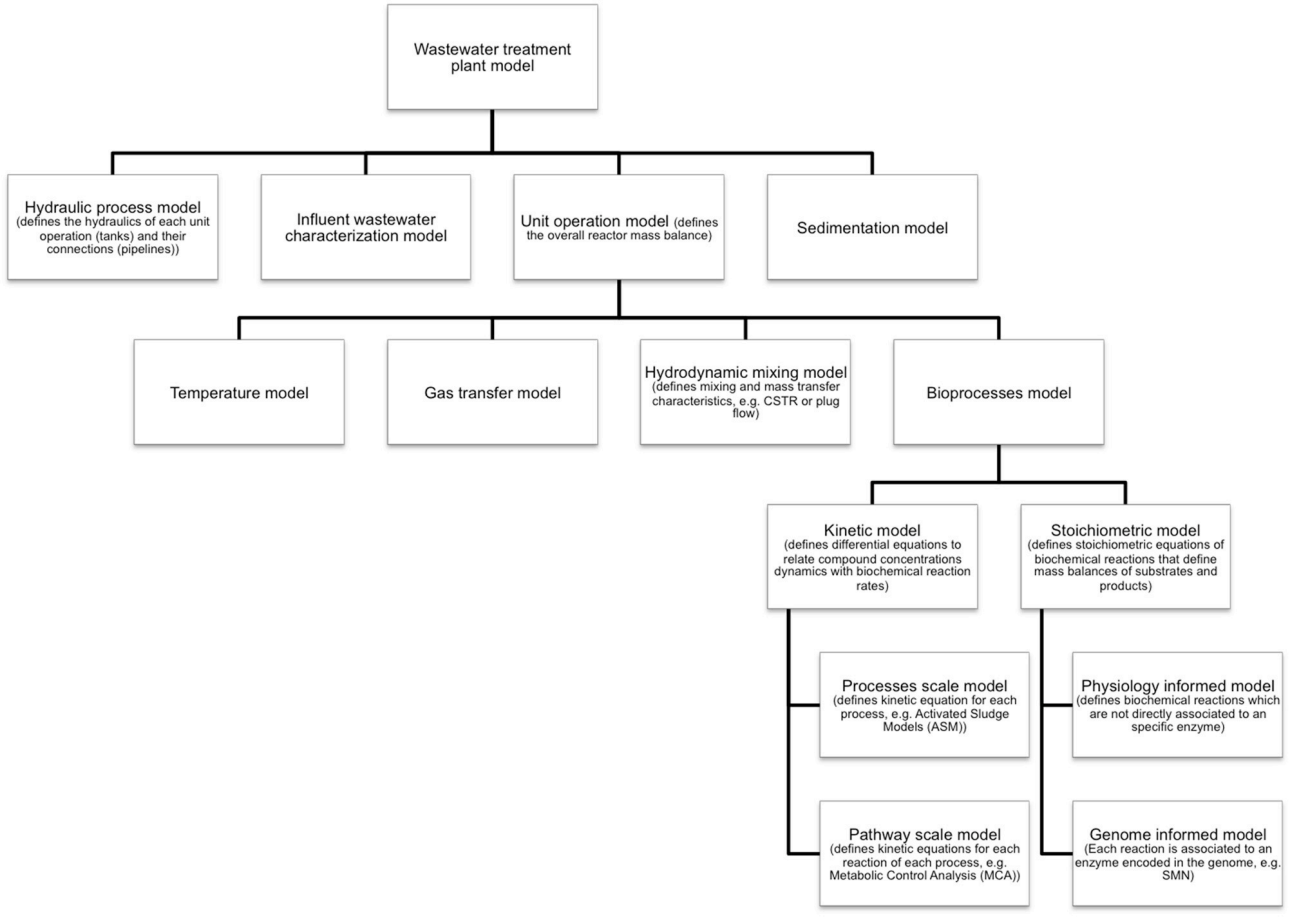
**Sub-models of an environmental system (e.g., a full scale wastewater treatment plant)**. SMN models are genome informed stoichiometric models of biological processes. Inherently, SMN is not a kinetic model therefore does not capture process dynamics. Nevertheless, SMN and kinetic models can be integrated in a common modeling framework.

Metabolic models are developed and applied when is necessary to account for detailed microbial physiology (Ishii et al., 2004). These models capture metabolic pathways as sequences of specific enzyme-catalyzed reaction steps converting substrates into cell products. Given this level of detail, metabolic models are commonly applied to (Oehmen *et al*., 2010): (i) generate mechanistic hypotheses from experimental observations; (ii) improve process efficiency by providing a quantitative basis for process design, control and optimization; (iii) estimate the activity of a specific microbial guild; and (iv) investigate the involvement of a specific metabolic pathway in observed processes. However, metabolic models capture only the molecular/biochemical aspect the whole environmental system. For example, a model of a full-scale wastewater treatment system implementing a biological treatment operation (e.g., activated sludge) has a hierarchy of sub models as shown in Figure 2 (Makinia, 2010). The diagram shows that metabolic models are extensions of bioprocess models and that also physical and chemical phenomenon such as hydrodynamics, mixing, temperature, and gas transfer have to be modeled in order to capture the full complexity of an environmental process. Nevertheless, each sub-model can operate as a standalone mathematical tool. In this sense, the inclusion of metabolic information is essential for deeper bioprocess understanding and operation improvement.

**Figure 3:**
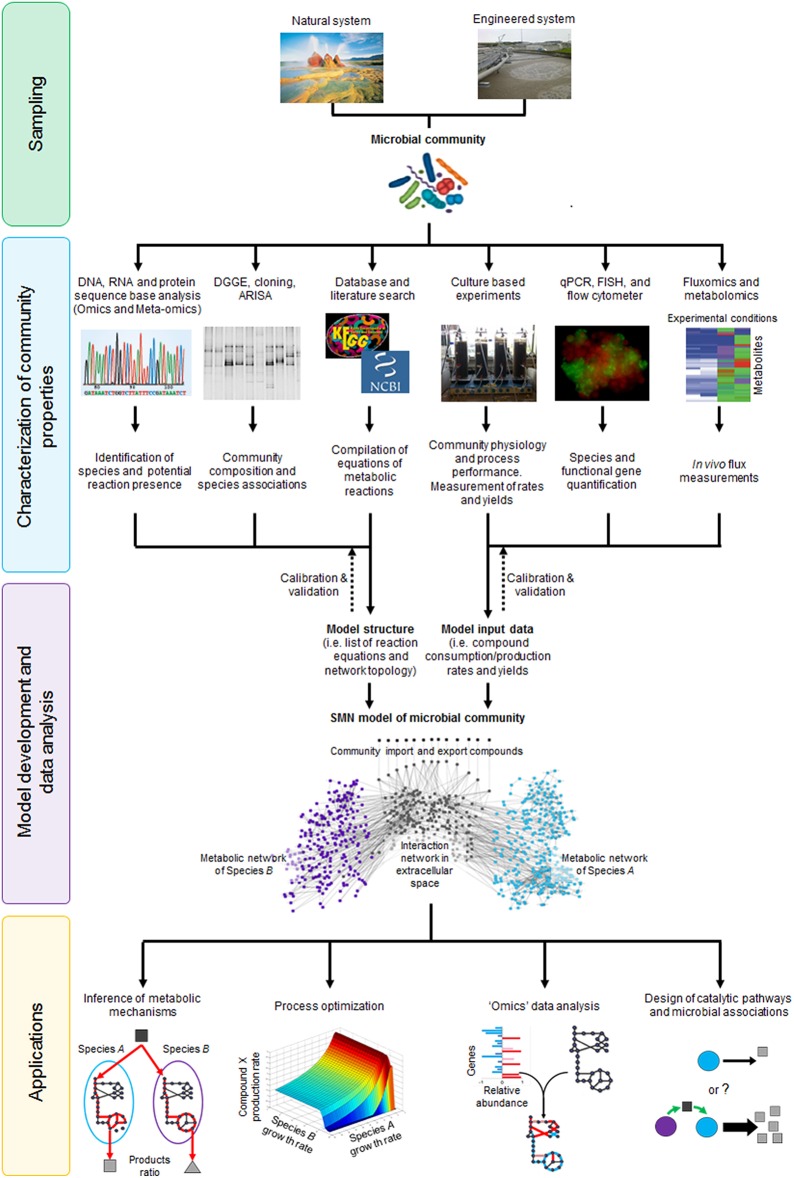
**The stoichiometric metabolic network modeling approach for analysis of microbial interactions and communities in natural and engineered environmental systems**. The approach is subdivided in four main stages (i) sampling of microbial communities from environmental systems; (ii) characterization of community properties and species interactions through culture dependent and culture independent techniques; (iii) integration of experimental data through model development and analysis; and (iv) application of SMN model as tool to study basic mechanisms or design processes. DGGE, Denaturing Gradient Gel Electrophoresis; ARISA, Automated Ribosomal Intergenic Spacer Analysis; qPCR, quantitative Polymerase Chain Reaction; FISH, Fluorescence *In-situ* Hybridization. Dotted lines represent rounds of model calibration and validation against experimental data. The artwork representing the “Microbial community” was taken from Vanwonterghem et al. (2014).

As shown in Figure 3, metabolic models serve as a bridge between molecular/biochemical research and environmental engineering practice, functioning as a tool that can better link the work of microbiologists and engineers in understanding and optimizing a particular environmental bioprocess (Oehmen *et al*., 2010). Several culture dependent and independent techniques can be applied to analyze community physiology (yields, growth rates, and metabolite consumption/production rates), ecology (species presence/absence and abundance) and molecular properties (functional gene, enzyme, and metabolite presence/absence and abundance). Data generated through these analyses can be encoded and integrated into a metabolic model of the original microbial community. The model is calibrated and validated by iteratively comparing model generated data against experimental data, which is a critical step to establish model reliability. Once that model accuracy is satisfactory, it can be applied to infer metabolic mechanisms, optimize processes, analyze high-throughput “omic” data or design novel catalytic pathways or microbial associations (Figure 3). Given that metabolic models both describe and quantify biological mechanisms, they are used as state-of-the-art research tools and for practical applications through linkages with bioprocess models.

### Introduction to SMN models

Stoichiometric metabolic network (SMN) models—also known in the literature as genome-scale or genome-informed metabolic (GEM or GIM) models—are mathematical representations of cell biochemistry used to quantify metabolic reaction rates and therefore describe cell phenotypes (Varma and Palsson, 1994b; Kitano, 2002; Ishii et al., 2004; Palsson, 2009). SMN models are data analysis tools particular to bioinformatics and systems biology disciplines. The aim of these disciplines is to investigate and understand the systematic relationships between genes, molecules, and organisms through computational modeling (Kitano, 2002; Kell, 2006; Park et al., 2008; Endler et al., 2009). SMN models have become an important tool for characterizing the metabolic activity of cells in biotechnological processes and have promising potential to assist in the analysis and understanding of microbial interactions (Lovley, 2003; Zengler and Palsson, 2012). The explosion in the number of new SMN models for up to 200 different organisms over the last few years highlights the increasing popularity of this approach, particularly in pharmaceutical and chemical industries (Feist and Palsson, 2008; Park et al., 2008; Milne et al., 2009; Kim et al., 2012; O'Brien et al., 2015). Although the method has inherent drawbacks and presents important challenges (please consult Section Applications of SMN Modeling of Microbial Interactions for further details), it continues to provide a fertile research field, as demonstrated by the recent growth of model analysis methods and tools (Durot et al., 2009; Kim et al., 2012; Lewis et al., 2012).

### Reconstruction of the metabolic network

Metabolic network are formulated using genomic information of the species to be modeled. For each microbial species or guild to be modeled, an initial metabolic network has to be formulated from gene-annotation data found in the scientific literature and online biochemical databases (e.g., KEGG, Model SEED, and NCBI, please consult Table 2 for details). These databases are used to obtain complete sets of stoichiometric equations to “map” or “reconstruct” complete biochemical pathways. Specialized biochemistry literature is also used to obtain details of reaction stoichiometry, cofactors and by-products. Usually information in the databases and the literature is incomplete (e.g., unknown reaction co-factors or compound synthesis steps). Therefore, therefore manual curation of the metabolic network—adding and balancing all equations to fill network gaps—is commonly necessary. The reader is referred to the excellent protocol by Thiele and Palsson (2010) for details of how to reconstruct metabolic networks for each species/guild to be modeled. Here, we provide a general description of the essential elements of the reconstruction process.

**Table 2:** **Examples of useful internet databases of biochemical reactions, metabolic pathways, and microbial genomes**.

**Database**	**Application**	**Internet URL**
KEGG. Kyoto Encyclopedia of Genes and Genomes	Very useful database with detailed information of enzymes, pathway reactions and compounds	http://www.genome.jp/kegg/
The Model SEED	Very useful database where complete genome scale models can be downloaded	http://seed-viewer.theseed.org/seedviewer.cgi?page=ModelView
NCBI. National Center for Biotechnology Information	Detailed information about literature, genomes, genes, proteins and compounds	http://www.ncbi.nlm.nih.gov/
BRENDA	Specific detailed information on enzymes and reactions	http://www.brenda-enzymes.info/
Metacyc	Specific detailed information of pathways and reactions	http://metacyc.org/
GOLD, Genomes On Line Database	Specific detailed information of genomes, genes	http://www.genomesonline.org/
BioModels database	Curated models of biological systems	http://www.ebi.ac.uk/biomodels-main/
BiGG database	Curated genome scale models	http://bigg.ucsd.edu/

*Please refer to Durot et al. (2009) or Thiele and Palsson (2010) for extensive lists of databases*.

**Figure 4:**
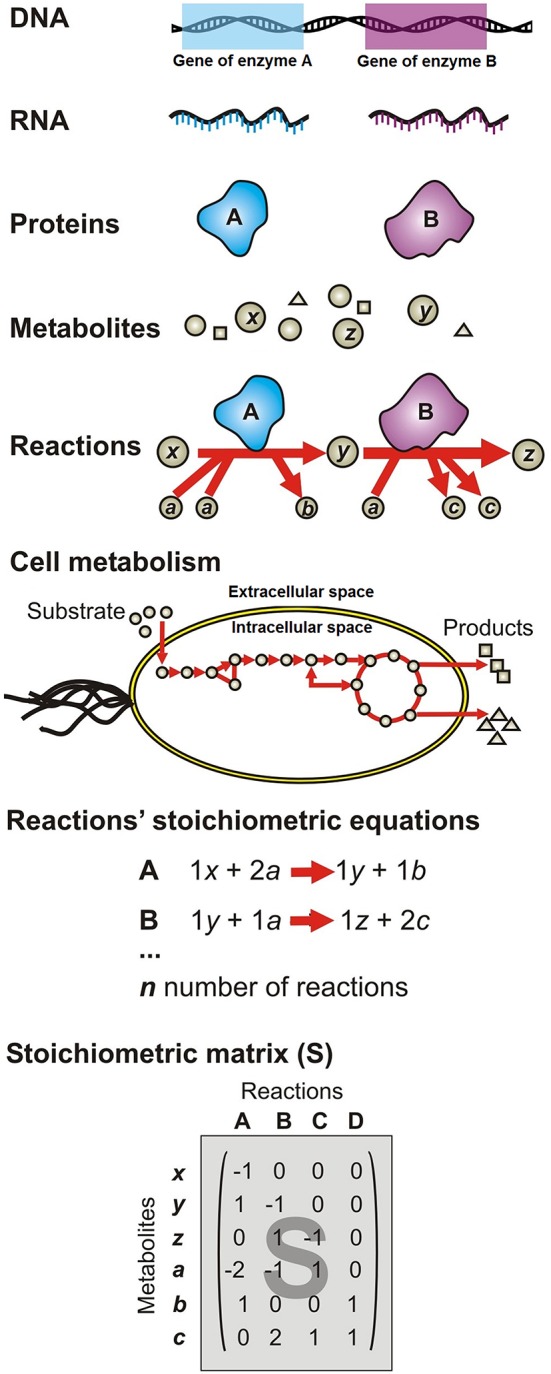
**Formulation of the stoichiometric metabolic network (SMN) of a single species (or microbial guild) using genomic information**. DNA encodes information to synthesize specific proteins with enzymatic activities (A and B); proteins catalyze specific reactions where metabolites are used as substrates (*x, a, y*) to be transformed into products (z, b, c); subsequent reactions form metabolic pathways, which constitute cell metabolism; each reaction is represented as a stoichiometric equation (A and B); the equations are then compiled in an extensive list of reactions involved in the modeled pathways.

A metabolic network consists of a list of mass and charge-balanced stoichiometric biochemical reactions that are classified as either reversible or irreversible (Savinell and Palsson, 1992; Thiele and Palsson, 2010). As illustrated in Figure 4, networks of biochemical reactions are reconstructed from existing knowledge of which genes are present in each species, as well as the function of genes. Figure 4 depicts the SMN reconstruction process as follows: the organism's DNA encodes information to synthesize specific proteins with enzymatic activities (A and B); proteins catalyze specific reactions where metabolites are used as substrates (*x, a, y*) to be transformed into products (z, b, c); subsequent reactions form metabolic pathways, which constitute cell metabolism; each reaction is represented as a stoichiometric equation (A and B); the equations are then compiled in an extensive list of reactions involved in the modeled pathways. The network topology concept refers to the web structure formed by metabolites interconnected through biochemical reactions, the biochemical pathway formed through these connections and how metabolites are distributed in different intracellular compartments (Varma and Palsson, 1994b; Orth et al., 2010).

Depending of the number of reactions they contain, SMN models may be classified as pathway-scale or genome-scale. Pathway-scale models contain reaction equations for specific and essential metabolic pathways. The size of this model ranges from 10 or 20 to 100 of equations, so they are easily developed, calibrated, and validated. In contrast, GEMs contain reaction equations for all metabolic pathways occurring in an organism, according to the catalytic enzymes encoded in its genome. The size of genome-scale models can go from a few hundred, for models of bacteria, and archaea with small genomes, to a couple of thousand for models of eukaryotic organisms (Kim et al., 2012). Although expansion of the model may improve the fitness to experimental data, it also presents important additional difficulties, such as: (a) the tendency to overestimate the rate of reactions in pathways that may only have a low flow of metabolites as SMN models assume that all enzymes in a pathway are present and active (unless explicit experimental evidence to the contrary); (b) the uncertainty of incomplete pathways or unknown reaction stoichiometry details, particularly in secondary metabolic pathways (Feist et al., 2009).

#### Compartmentalization and reaction equations of the metabolic network

The reactions can be modeled as occurring in different cellular compartments such as the periplasm, chloroplast, cytoplasmic and extracellular spaces (Chain et al., 2003). To do this, labels such as [e], [p], and [c] are assigned to metabolic compounds to indicate their occurrence in a particular compartment. The metabolite “x[e]” is thereby differentiated from “x[p]”and their diffusion between two different compartments is defined as “x[e] ↔ x[p].” By using compartmentalization, the reconstructed networks can have stoichiometric equations to represent three types of biochemical reaction (Thiele and Palsson, 2010): (i) Exchange reactions, which define the composition of the “synthetic growth medium.” They do not represent a biochemical conversion but rather define which chemical compounds are consumed into or exported from the metabolic system; (ii) Transport or diffusion reactions, which define a compounds flow of mass from one compartment to another; and (iii) True metabolic reactions (inferred from organism genomes), which define biochemical transformations of compounds to form other compounds catalyzed by an enzyme. Network reactions can be defined as reversible or irreversible reactions depending on the catalytic enzyme properties. Additionally, identical or similar reactions—including reversible and irreversible versions of the same reaction—can be included in the network because each version might be associated with a different enzyme or set of genes.

### Conversion of reconstructed SMN into a mathematical model

The conversion of an organism or microbial guild SMN reconstruction into a model requires transformation of the reaction list into a mathematical matrix format. Thus, the equations' stoichiometric coefficients are arranged in the stoichiometric matrix (*S*), of size *m* per *n*. Every row of this matrix represents a unique compound *i* (for a system with *m* number of compounds); and every column represents a reaction *j* (for a system with *n* number of reactions) (Figure 4). So that, an entry *s*_*ij*_ in the matrix *S* is a stoichiometric coefficient of metabolite *i* in reaction *j*. A negative entry on the *S* matrix indicates that the corresponding compound is consumed in the reaction. Conversely, a positive entry indicates that the corresponding compound is produced in the reaction. A stoichiometric coefficient of zero is used for every metabolite that does not participate in a particular reaction. The *S* matrix contains all the information relating to the reactions modeled for a particular organism (Varma and Palsson, 1994a; Orth et al., 2010). The modeled system boundaries are defined using physicochemical and environmental data as network input data (constraints) (Varma and Palsson, 1994a). The constraints can be grouped into any one of five categories (Price et al., 2003; Oberhardt et al., 2009): (i) physicochemical (e.g., conservation of mass defined in the *S* matrix); (ii) topological (e.g., compartmentalization and spatial restrictions associated with metabolites/enzymes defined in the *S* matrix); (iii) genotypical (defined by the profile of functional genes expressed by the organisms under a given environmental condition which in turn defines which reactions allow the flow of metabolites) (iv) environmental (i.e., media composition; these constraints are captured in the model as lower (α_*j*_) and upper (β_*j*_) bounds of substrate consumption rates; and (v) thermodynamic (defined by the observed compounds concentration and fluxes as well as Gibbs energy of reaction, then captured in the model as reaction reversibility). As shown in Figure 3, physicochemical, topological, and genotype constraints generally define the structure of the model (i.e., the list of equations and network topology), while environmental and thermodynamic constraints generally define model input data. In the same way that a cell is unique in having one genome and many phenotypes, a metabolic reconstruction is unique for its target organism but context-specific models can be derived by changing the constraint values, therefore representing cellular functions under different environmental or state conditions (Thiele, 2009).

### Model simulation

Once the metabolic network is captured in a matrix format, different mathematical analyses can be performed. These computational methods have been reviewed in recent publications such as Durot et al. (2009), Kim et al. (2012), and Lewis et al. (2012). Lewis et al. (2012) presents a comprehensive overview of the different methods and their applications. In general, the methods (algorithms) to simulate SMN models follow two main categories, biased methods formulated as optimization problems and unbiased methods formulated to characterize all possible solution able to be obtained given the network topology (characterize network's solution space) (Schellenberger and Palsson, 2009). Hence, biased methods include the optimization of an objective function to identify physiologically relevant flux distributions; and unbiased methods describe all possible network's flux distributions. FBA is the most basic and commonly-used biased method for simulating SMN models. It is effective in making quantitative predictions of flux distributions using a few governing constraints on the model (Edwards et al., 2001; Oberhardt et al., 2009; Orth et al., 2010). A complete description of the plethora of these methods and their applications is beyond the scope of this review and will not be discussed further. Nevertheless, an introductory description of FBA is provided below to illustrate how metabolic reaction rates (a.k.a. fluxes) are predicted using SMN models.

#### Flux balance analysis (FBA)

FBA and their related methods are used to predict steady state fluxes (i.e., reaction rates) in the metabolic network, rather than time-dependent metabolite concentrations (Varma and Palsson, 1994a). FBA provides a “snapshot” estimation of the rates of all network reactions simultaneously operating under a specific environmental or physiological state (Varma and Palsson, 1994b; Orth et al., 2010). A set of specific reaction rates estimated at a specific steady state is called flux distribution (*v*) (Varma and Palsson, 1994b; Orth et al., 2010). All reaction rates are generally expressed in units of millimoles (mmol) of compound produced or consumed per unit of biomass per hour (h). Biomass is commonly expressed as grams of dry weight (gDW)—although, for microbial community models can also be expressed as grams of volatile suspended solids (gVSS) or chemical oxygen demand (gCOD)—so that the reaction rates have the following units:

$${\text{mmolg DW}}^{-1}{\text{ h}}^{-1}$$

In FBA the dynamic mass-balance for each compound in the network is represented by the equation:

(1) $$\begin{array}{l} \frac{dX}{dt}=S•v \end{array}$$

where *X* is the vector of metabolite concentrations, *t* is time, *S* is the stoichiometric matrix with size *m* × *n* and *v* is the vector of metabolic fluxes through all network's reactions (Savinell and Palsson, 1992). Transitions of metabolic activity are typically on the order of a few minutes, which is much faster than cellular growth rates and other changes in the microorganisms' environment. So that metabolic changes are considered to be in a steady-state relative to growth and environmental transients (Varma and Palsson, 1994a). In a system at steady-state the change in concentration of metabolites over time is equal to zero so that:

(2) $$\begin{array}{l} \frac{dX}{dt}=S•v=0 \end{array}$$

Equation (2) implies that the fluxes of metabolic compound formation *i* are balanced with its degradation fluxes so that the sum of fluxes equals zero. In other words, metabolite accumulation is disregarded and all the flux of mass entering into the network goes out.

A precise definition of the boundary of the system to be modeled is also needed to formulate an explicit mathematical representation. Consequently, a specific environmental or phenotypic condition (described in Section Conversion of Reconstructed SMN into a Mathematical Model) is modeled by defining specific values of reaction rates using the following form:

(3) $$\begin{array}{l} \alpha_{j}\leq v_{j}\leq \beta_{j} \end{array}$$

where α_*j*_ and β_*j*_ represent the lower and upper bounds for reaction rate *v*_*j*_. A lower bound (α) and an upper bound (β) are in fact set to every reaction. Bounds for exchange reactions represent the flow of nutrients into and out of the biochemical system; while bounds for transport reactions (occurring across cell and subcellular compartment membranes) and metabolic reactions (occurring within the confines of the cell membrane) represent physicochemical constraints on reaction rates due to thermodynamics or catalytic enzyme availability (Orth et al., 2010). The set rates define a given environmental condition and physiological state, and reduce (or “constraint”) the number of possible solutions for *v* (Edwards et al., 2001; Oberhardt et al., 2009; Orth et al., 2010).

As SMNs (represented as *S*) are underdetermined systems, meaning that there are more reactions than there are compounds (*n* > *m*), there is no unique flux distribution (*v*) solution. An optimization algorithm [linear programing (LP) for FBA] is therefore used to find the optimal *v* that minimizes or maximizes a particular objective function (Z) defined by the user (Varma and Palsson, 1994a). Typically, the objective function is set to maximize the rate of the biomass production reaction (*Z* = *v*_*biomass*_), although other objective functions, such as minimization of resource utilization and maximization of ATP production can be used depending on the simulation condition (Schuetz et al., 2007). The output of FBA is a particular vector *v* that maximizes or minimizes *Z* (Oberhardt et al., 2009; Orth et al., 2010). The mathematical formalism for FBA's optimization problems is as follows:

$$\begin{array}{l} max\left(v_{Biomass}\right)\\ s.t.\left\{\begin{array}{l} \text{S}•v=0\\ \alpha_{j}\leq v_{j}\leq \beta_{j}\\ v_{Substrate\;Uptake}=Substrate\;uptake\;\\ measured\;experimentally \end{array}\right\} \end{array}$$

Biomass production is mathematically represented by adding an artificial “biomass formation equation.” The equation defines precursor metabolites at a stoichiometry that simulates the production of one gram of biomass dry weight (DW) (Pramanik and Keasling, 1997). To formulate an equation for biomass production, the dry weight cellular composition of the organism of interest, and its energetic requirements for biomass synthesis need to be obtained experimentally, from the literature, or estimated using data from phylogenetically related organisms (Feist and Palsson, 2010). Cellular composition refers to the fraction of proteins, RNA, DNA, carbohydrate, lipids, polyamines, and other biomass constituents. These components are enlisted in the biomass reaction as constituent metabolites such as amino acids, nucleic acids, etc. Stoichiometric coefficients of enlisted metabolites are scaled to satisfy the required mass to form one gram of biomass dry weight. As a result, the flux through the biomass reaction (in mmol gDW^−1^ h^−1^ units) is equivalent to the growth rate (μ, in h^−1^ units) of the organism as gDW and mmol units can be eliminated (Oberhardt et al., 2009; Feist and Palsson, 2010).

### Computational tools and software

**Figure 5:**
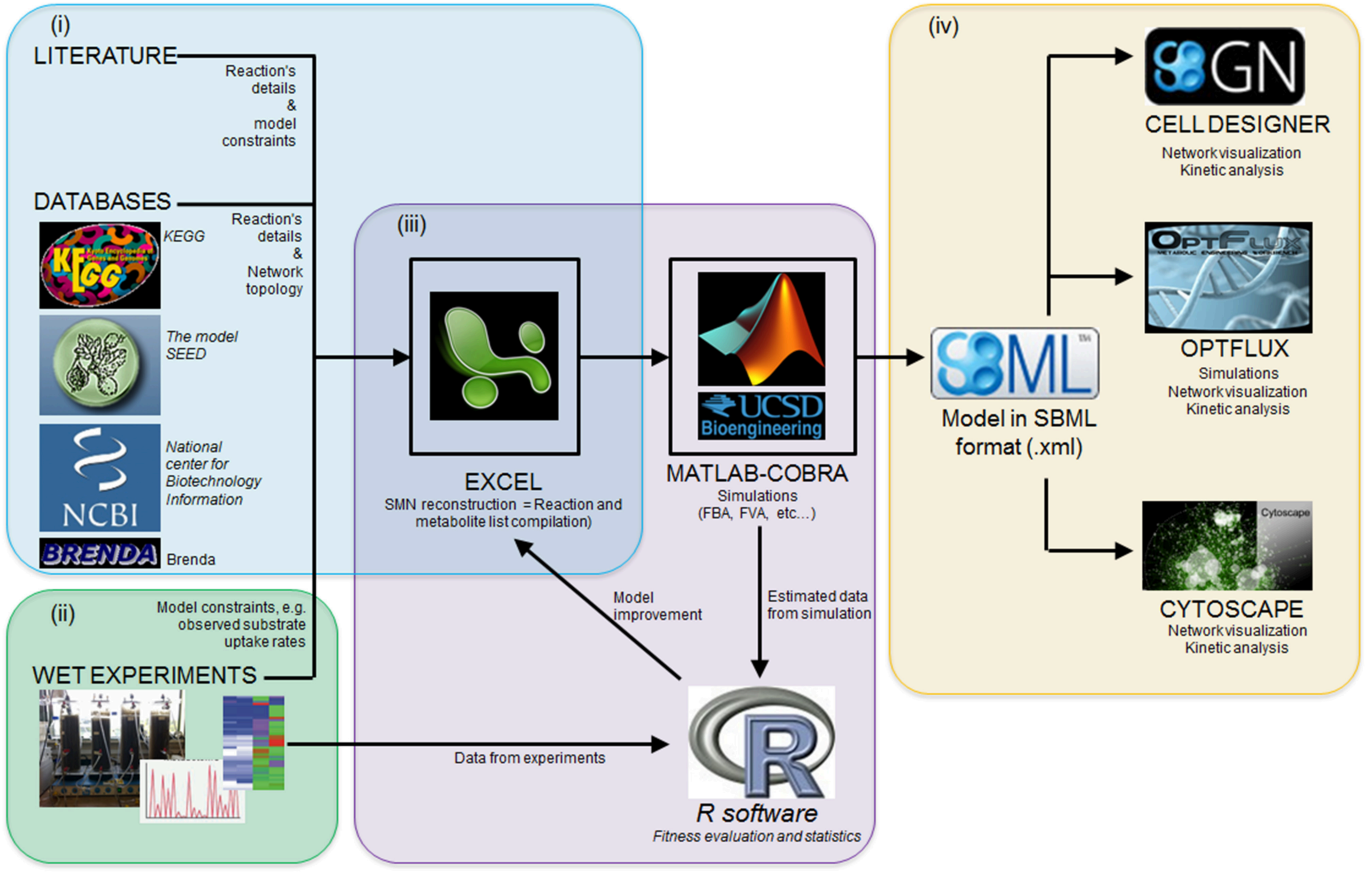
**A research workflow to model microbial interactions using SMN models**. (i) network reconstruction step; (ii) acquisition of experimental data; (iii) the model calibration step, which involves the statistical comparison of model estimated data against experimentally observed data and further model parameter adjustment to improve predictions; (iv) the calibrated model can be used to perform further analysis in other software platforms. See Section Computational Tools and Software for details.

Several software packages are used to build and simulate SMN models. Table 3 lists some of these software packages and provides details of their application. The reader is referred to Medema et al. (2012) for an extensive review of computational tools and software packages for metabolic network modeling. The majority of these packages are available in the internet as freeware. Figure 5 illustrates the research workflow that we, the authors, commonly adopt to perform modeling studies. The corresponding data resources and the software packages used for each step are also shown. In step (i), searches of scientific literature and biochemical databases are undertaken to acquire stoichiometric equations of biochemical reactions forming specific metabolic pathways. The network is reconstructed in a spreadsheet as this format is easy to use and the data can easily be transferred among different simulation software. The list of equations is loaded into MATLAB® (The MathWorks, Inc., Natick, Massachusetts, United States.) using the COBRA toolbox. Model loading and simulation can be also done in software packages such as Optflux (Rocha et al., 2010). In the following step, (ii), Excel and R software are used to record the metabolite concentration curves observed in experimental cultures and then calculate specific rates for the production and consumption of culture substrate and products. Acquisition of experimental data characterizing the microbial community properties (step ii, also shown as stage ii in Figure 3) is crucial as such data is required to compare all model generated predictions. In step (iii), specific rates of substrate consumption measured in experimental cultures are used as SMN model input data (constraints) in the MATLAB-COBRA toolbox. The model is simulated (e.g., via FBA) and fitted (calibrated) to datasets observed from cultures (Perez-Garcia et al., 2014a). In step (iv), once the model is calibrated, the COBRA toolbox is used to perform a second round of model analysis to estimate metabolic rates; and the effect of operational parameters of experimental cultures on metabolic pathways is inferred from these estimated metabolic rates (stage “Applications” of Figure 3). Finally network visualization and network topology analysis can be performed using Cytoscape, CellDesigner, and Optflux (consult Table 3 for further software information). It is important to recognize that SMN models are not stand alone tools but rather support tools to be used to analyze data, generate hypotheses and design new “wet” experiments.

**Table 3:** **Examples of software packages used to develop and simulate SMN models**.

**Software package**	**Application and software type**	**Internet URL for download**
Microsoft Excel	Build-up of SMN reconstruction file. Standalone software	http://office.microsoft.com/en-us/excel/
MATLAB®	Software and computing environment	http://www.mathworks.com/
	Standalone software	
COBRA toolbox	SMN modeling and simulation in MATLAB	http://opencobra.sourceforge.net/openCOBRA/Welcome.html
	Free MATLAB toolbox	
Optflux	SMN modeling and simulation. Standalone and free software	http://www.optflux.org/
FASIMU	SMN modeling and simulation. Standalone and free software	http://www.bioinformatics.org/fasimu/
SBML toolbox	Functions allowing SBML models to be used in different modeling software	http://sbml.org/Software/SBMLToolbox
	Free toolbox for modeling software	
libSBML 5.5.0	Programming library to manipulate SBML files	http://sbml.org/Software/libSBML
	Software library	
GLPK solver	Optimization problem solver	http://www.gnu.org/s/glpk/
Tomlab solver	Optimization problem solver	http://tomopt.com/tomlab/
Gurobi solver	Optimization problem solver	http://www.gurobi.com/
Cytoscape	Network visualization	http://www.cytoscape.org/
	Stand alone and free software	
CellDesigner	Pathway graphic reconstruction. Standalone and free software	http://www.celldesigner.org/
anNET	Analysis of metabolites concentrations with SMN models. Free MATLAB toolbox	http://www.imsb.ethz.ch/researchgroup/nzamboni/research

## Approaches to modeling microbial interactions using SMN models

While several aspects of microbial metabolism can be fruitfully addressed by studying pure cultures of individual microbial species, many environmental bioprocesses require an understanding of how microbes interact with each other (Lovley, 2003; Klitgord and Segrè, 2010; Zengler and Palsson, 2012). A lack of information about environmental factors controlling the growth and metabolism of microorganisms in natural and polluted environments often limits the implementation of monitoring or management strategies (Lovley, 2003). Within this context, SMN modeling can be a relevant computational approach to underpin the analysis of microbial interactions in such processes (Miller et al., 2010; Vilchez-Vargas et al., 2010). The development of SMN modeling methods capturing species interactions enables increasingly realistic predictions of whole community phenotypes (Stolyar et al., 2007; Oberhardt et al., 2009) and quantification of rates of exchange of compounds between different populations (Lovley, 2003; Stolyar et al., 2007). An important advantage of using SMNs is that many different types of metabolic interaction occurring simultaneously can be modeled. For instance, it is possible to model two species simultaneously competing for several nutrients (e.g., oxygen, phosphate, and carbon dioxide in nitrification systems) but having a commensal interaction through other compounds (e.g., nitrite in nitrification systems). In addition, a relevant feature of SMN models is that they can be applied to simulate the cellular metabolism of homogenous mixtures of suspended cells such as those in stirred tank reactors, as well as in biofilms or stratified systems by implementing appropriate reaction–diffusion equations (Rodríguez et al., 2006). Given that SMN models contain extensive details of many metabolic pathways and intermediates, the exchange of multiple metabolites between different species can be analyzed.

**Figure 6:**
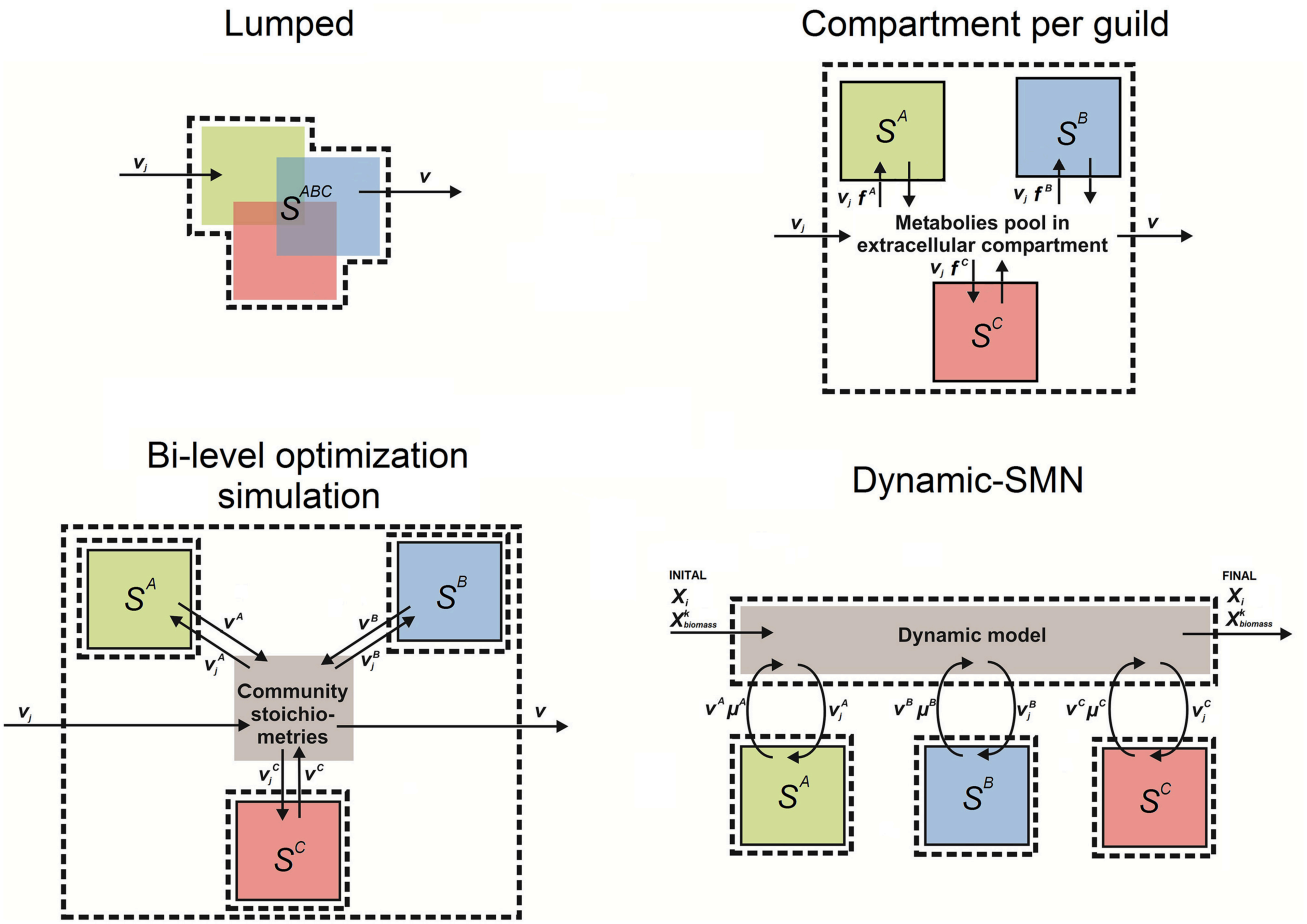
**Conceptual scheme of the four approaches to model mixed microbial cultures using stoichiometric metabolic networks**. In all figures boxes S^*A*^, S^*B*^, S^*C*^ represent sets of equations (captured as an *S* matrix) of metabolic reactions occurring in organisms/guilds *A, B*, and *C*, respectively. *S*^*ABC*^ is a matrix lumping metabolic reactions occurring in organisms/guilds *A, B*, and *C*. These sets of reactions can have any number of sub compartments to model reactions occurring in the extracellular space and organelles; boxes with dashed lines indicate model (system) boundaries; boxes with solid lines indicate guild boundaries; *v*_*j*_ is the flux of metabolite in reaction *j*; *V* is the vector of fluxes estimated by the model; *V*^*k*^ is the vector of fluxes estimated by the model of species/guild *k* (*A, B*, or *C*); *X*_*i*_ is the concentration of metabolite *i*; μ^*k*^ (a.k.a. $v_{biomass}^{k}$) is the growth rate (biomass production rate) of species *k*; *f*^*k*^ is the fraction of species *k* in community's biomass; and $X_{biomass}^{k}$ is the biomass concentration of modeled species/guild *k* (*A, B*, or *C*). Figure inspired in Taffs et al. (2009) modeling approaches diagrams.

SMN modeling approaches have been used since 1999 to understand the behavior of biological systems in complex environments and to model organisms relevant to environmental bioprocesses, when Pramanik et al. (1999) first developed a SMN model of phosphate accumulating organisms. This was the first attempt to adapt SMN's to model microbial communities. Later Lovley (2003) presented a coherent framework to combine omic techniques, computational biology, and metabolic network modeling to study environmental processes. As shown in Table 4, the literature to date indicates that SMN modeling has been applied to quantify metabolic rates in environmental bioprocesses in only a few studies. Generally, in these studies the number of species in the modeled community is referred to as *N* while each modeled species is referred to as *k* (Zomorrodi and Maranas, 2012). Table 4 also shows that four approaches have been developed to model microbial interactions in environmental processes using SMNs. We define these approaches as: (i) lumped networks, (ii) compartment per guild networks (also known as multi-compartment networks), (iii) dynamic-SMN (also known as hybrid SMN), and (iv) bi-level optimization simulation. These approaches are described in the following sections; a conceptual scheme for each modeling approach is illustrated in Figure 6.

**Table 4:** **Approaches and applications for SMN modeling of environmental bioprocesses**.

**Modeling approach**	**Environmental bioprocess**	**Modeled organisms/guilds**	**Model application**	**References**
Lumped network	Enhanced biological phosphorus removal (EBPR)	Mixed population of Phosphate accumulating organisms	Description of how carbon, energy, and redox potential are channeled through metabolic pathways	Pramanik et al., 1999
	Nitrification	*Nitrosomonas* sp. and *Nitrobacter* sp.	Description the redox reactions of the electron transport chain	Poughon et al., 2001
		*Nitrosomonas europaea*	Quantification of rates of N_2_O production through diverse pathways	Perez-Garcia et al., 2014a
	Anaerobic fermentation of carbohydrates to alcohols and carboxylic acids	Mixed population of anaerobic fermentative organisms	Link of operation parameters (feeding composition, gas partial pressure and pH) to product formation	Rodríguez et al., 2006
	Photoautotrophic growth of planktonic/suspended cells	*Synechocystis* sp. PCC 680, single species model	Description of functional properties of phototrophic growth	Knoop et al., 2010, 2013; Maarleveld et al., 2014
			Description of photosynthetic process under different lights and inorganic carbon concentrations	Nogales et al., 2012
		*Cyanothece* sp. ATCC 51142 single species model	Study of photosynthesis activity in the electron transport chain	Vu et al., 2012
		*Chlamydomonas reinhardtii*, single species model	Study of biological photosynthesis and phototaxis processes	Chang et al., 2011; Hong and Lee, 2015
			Description of metabolic regulation of mixotrophic growth,	Chapman et al., 2015
	Phototropic growth of microbial mat and toxin production	*Synechococcus* spp., *Chloroflexus* spp, and sulfate reducing bacteria	Description of metabolic mechanism behind the observed biomass productivity, relative abundance and toxin productivity	Taffs et al., 2009
	Subsurface anaerobic fermentation of organic matter	*Clostridium cellulolyticum, Desulfovibrio vulgaris*, and *Geobacter sulfurreducens*	Description of substrate consumption routes in microbial community	Miller et al., 2010
	Fermentative anaerobic production of H_2_ and acetate	Community enriched with *Clostridium* sp., *Lactobacillus* sp., and *Seleomanas* sp.	Description of metabolic routes for product degradation	Chaganti et al., 2011
	Waste sugars fermentation to PHA	Mixed population of PHA producing organisms, Lumped-dynamic model	Linking operation parameters (feeding regime) to product formation	Dias et al., 2008
			Linking operation parameters (feed composition) to product formation	Pardelha et al., 2012
			Assessing single population contribution to process performance	Pardelha et al., 2013
Compartment per guild network	Methanogenic fermentation	*Desulfovibrio vulgaris* and *Methanococcus maripaludis*	Description of metabolic mechanism behind the association of organisms	Stolyar et al., 2007
		*Geobacter metallireducens* and *Geobacter sulfurreducens*	Investigation of synthrophic associations for direct interspecies electron transfers	Nagarajan et al., 2013
	Growth of phototropic microbial mat	*Synechococcus* spp, *Chloroflexus* spp, and sulfate reducing bacteria	Description of metabolic mechanism behind the observed biomass productivity, relative abundance, and toxin productivity	Taffs et al., 2009
	Commensalism and mutualism between pairs of organisms	Pairs of seven different bacteria^+^	Novel process development. Identifying new environmental conditions that support specific ecological interactions	Klitgord and Segrè, 2010
	Nitrification	Four AOB species together with four NOB species^*^	Description of metabolic mechanisms of NO and N_2_O turnovers during biological nitrogen removal	Perez-Garcia et al., 2016
	Syntrophic consortium	*Escherichia coli* strains consortium	Prediction of species abundances and metabolic activities. Analysis of global responses to metabolic limitations	Khandelwal et al., 2013
Dynamic-SMN	*In situ* uranium bioremediation by microbial reduction and precipitation	*Geobacter sulfurreducens*	Description of metabolic mechanisms in ground water bodies, Hydrodynamic-SMN model	Scheibe et al., 2009
	*In situ* uranium bioremediation by microbial reduction and precipitation	*Geobacter sulfurreducens* and *Rhodoferax ferrireducens*	Description of metabolic mechanisms in ground water, Monod-SMN	Zhuang et al., 2011
	Nitrification	An AOB species together with an AOB species	Investigation of mechanisms causing discrepancies between functional and composition changes in communities	Louca and Doebeli, 2015
Bi-level simulation	Phototropic growth of microbial mat	*Synechococcus* spp, *Chloroflexus* spp, and sulfate reducing bacteria	Description of metabolic mechanism behind the observed biomass productivity, relative abundance and toxin productivity	Taffs et al., 2009
		*Synechococcus* spp, *Chloroflexus* spp, and sulfate reducing bacteria	Assessing the effect microbial community structure on the total community biomass	Zomorrodi and Maranas, 2012
	Methanogenic fermentation	*Desulfovibrio vulgaris* and *Methanococcus maripaludis* synthorphic association	Linking the effect of microbial community composition to process performance.	Zomorrodi and Maranas, 2012
		*Simithella* spp., *Desulfovibrio* spp, *Methanocalculus* spp., *Methanoculleus* spp., *and Methanosaeta* spp.	Assessing the impacts of metabolic redundancy in microbial communities. “Meta-omics” data analysis	Embree et al., 2015
	Subsurface anaerobic fermentation of organic matter	*Clostridium cellulolyticum, Desulfovibrio vulgaris*, and *Geobacter sulfurreducens*	Description of substrate consumption routes in microbial community	Zomorrodi and Maranas, 2012

+ *Genome scale models of Escherichia coli, Helicobacter pylori, Salmonella typhimurium, Bacillus subtilis, Shewanella oneidensis, Methylobacterium extorquens, and Methanosarcina barkeri*.

* *AOB, ammonia oxidizing bacteria: Nitrosomonas europaea, Nitrosomonas eutropha, Nitrosospira multiformis, and Nitrosococcus oceani. NOB, nitrite oxidizing bacteria: Candidatus Nitrospira defluvii, Nitrobacter winogradskyi, Nitrobacter hamburgensis, Nitrospina gracilis*.

### Lumped network approach

Here, the community is modeled as a single entity in which all metabolic reactions and metabolites from the species/guilds are combined into a single set of reactions (i.e., a single *S* matrix) (Figure 6). A metabolic network of the whole mixed microbial population is built up by inventorying the most common catabolic reactions, i.e., electron transport chain, glycolysis, tricarboxylic acid cycle (TCA), and amino acid synthesis; and later adding reactions of pathways unique to specific species. For instance, Figure 6 depicts that reactions from the species A, B, and C belong to a single set of equations (system), where overlapped blocks represent common reactions. Reactions of discrete pathways can be lumped into a single reaction that represents the overall pathway (Rodríguez et al., 2006). Reactions catalyzed by more than one species/guild are only considered once, therefore pathway redundancy is disregarded. The spectrum of compounds produced by the community is generally obtained by maximizing the rate of the reaction that represents the overall production of biomass by the community, which sums all biomass precursors (*i*) with stoichiometric coefficients (*s*) synthetized by *N* number community members (*k*) (i.e., $Z=max\left(v_{biomass}^{community}\right)$ where $v_{biomass}^{community}=\sum_{k=1}^{N}s_{i}^{k}$). The lumped approach models the community metabolic potential by treating all enzymatic activities and metabolites as residents of the same physical space, therefore intracellular compartments are commonly neglected.

The approach is based on the assumption that all the organisms in the community have reactions in common and exploit the environment in a similar way (Table 4). It treats the microbial community as a single virtual microorganism catalyzing common biochemical pathways (Rodríguez et al., 2006). The virtual microorganism should be regarded as a representation of the different microbial strains involved in the bioprocess. This assumption brings both advantages and disadvantages. Ignoring microbial diversity and assuming a virtual microorganism able to carry out the most common biological conversions is acceptable in steady state and completely mixed conditions (Rodríguez et al., 2006) thus, simplifying the processes of model development and calibration. The metabolic potential based on community functional gene annotations can be directly investigated, as the assignment of each reaction to a constituent guild is unnecessary. The approach is quite flexible, can be scaled to different levels of detail and has low computational burden. With these advantages, the method is uniquely suited for initial and exploratory analyses of poorly understood communities (Taffs et al., 2009). Nevertheless, a minimum knowledge of community metabolism and physiology is required. In other hand, a major disadvantage is that microbial diversity and the dynamics of the process are neglected using the lumped approach (Rodríguez et al., 2006). Consequently, lumped networks capture the overall matter and energy transformations catalyzed by the community without providing detailed information of individual guilds and their interactions (Taffs et al., 2009). This method also neglects the logistics associated with transferring metabolites between organisms, including conversion of the relevant metabolite into one for which transporters are available (Taffs et al., 2009). Consequently, the lumped network approach is not strictly suitable to model microbial interactions but rather to analyze the overall behavior of a given community.

### Compartment per guild approach (multi-compartment)

In a compartment per guild network, each organism or guild is modeled as a distinct compartment of the network and exchangeable metabolites are transferred through an extra compartment representing the extracellular environment (Stolyar et al., 2007; Taffs et al., 2009; Klitgord and Segrè, 2010) (Figure 6). This fictitious extra compartment represents the extracellular environment shared by the microbial species/guilds so that they are modeled as being spatially separated by the extracellular medium (Klitgord and Segrè, 2010). The approach is implemented by assigning reactions and metabolites to a network representing each guild, with suffixes on metabolite identifiers preventing sharing of compounds common to the metabolism of multiple guilds. For instance, ammonium (NH${}_{4}^{+}$) is a metabolite for species/guilds A and B, so that in the model the ammonium entity is defined three times, one for each species [e.g., NH${}_{4}^{+}$(a) for species/guild A and NH${}_{4}^{+}$(b) for species/guild B] and an extra one for the shared extracellular environment [e.g., NH${}_{4}^{+}$(e)]. Explicit transport reactions are defined to account for the exchange of metabolites between species/guild members and the extracellular environment (Taffs et al., 2009). This allows interactions such as the commensalism and competition to be captured (Figure 1). Biomass formation reactions are defined for each modeled species/guild. The optimization problem is generally solved by maximizing the production rate of the community biomass, which sums the rates of biomass production of all modeled species (i.e., $Z=\max\left(v_{biomass}^{community}\right)$, where $v_{biomass}^{community}=\sum_{k=1}^{N}v_{biomass}^{k}$). Species abundances can be captured by scaling the overall community substrate uptake rates with a vector containing the fractions of species *k* in the biomass (*f*^*k*^), $v_{substrate\;uptake}^{k}=v_{substrate\;uptake}^{community}*f^{k}$. Consequently species substrate uptake rates are proportional to species biomass abundance.

The compartment per guild approach allows tracing of the metabolic behavior of each modeled species/guild. Dividing the community into species/guild-level compartments linked by transferred metabolites, e.g., oxygen, is an intuitive way to represent interactions within a community. This approach is optimal to analyze pairwise interactions in communities of only two different microbial guilds. Examples of such small communities are the ones formed by ammonia and nitrite oxidizing bacteria in nitrification processes (Ferguson at al., 2007) or the ones formed by microalgae and plant growth promoting bacteria (de-Bashan et al., 2005; de-Bashan and Bashan, 2010). It is also an ideal method for understanding which guild performs a particular metabolic transformation. For example, it is easy to estimate the fraction of total biomass or ATP produced by each guild (Taffs et al., 2009). Also, the approach allows the capturing species abundance profiles, as observed in experiments (Perez-Garcia et al., 2016). Separating species/guild metabolism in different compartments makes it possible to verify potential microbial interactions, or to formulating new growth media on basis of each species metabolic requirements (Klitgord and Segrè, 2010). A drawback of this approach is that the size of the resulting network can lead to a “combinatorial explosion” of new pathways composed by reactions from different guilds (Klamt and Stelling, 2002). To address this limitation, the models for each guild member can be constructed to only capture the necessary metabolic capabilities while maintaining computational tractability (Taffs et al., 2009). A second drawback of this approach is the requirement for significant *a priori* information or assumptions, as specific transport reactions must be assigned to each individual species/guild (Stolyar et al., 2007).

### Bi-level optimization approach

The approaches described above rely on either a single objective function to describe the entire community (Stolyar et al., 2007) or separate optimization problems for each microorganism (Tzamali et al., 2011). Bi-level optimization integrates both species and community-level fitness criteria into a multi-level/objective framework. The bi-level optimization approach is based on the assumption that a universal community-specific fitness criterion does not exist (Zomorrodi and Maranas, 2012). This approach uses successive rounds of simulations to analyze potential interactions within a community. A first round of optimization simulations (e.g., flux balance analysis) is applied to each modeled guild in isolation. Then the output data is mined for ecologically relevant interactions, compiled and used to define new stoichiometric reactions that are used in a second round of optimization simulations to examine the potential for interactions between guilds (Figure 6). Conceptually, the first round of simulations provides guild-level compound production rates, then proportions between estimated rates define new community stoichiometry's which are then used to define inter-guild interactions (Taffs et al., 2009). The bi-level optimization approach, like that developed as the OptCom algorithm (Zomorrodi and Maranas, 2012), postulates a separate biomass maximization problem for each species as initial (inner) optimization problems, consequently capturing driving forces of species-level fitness. Inter-guild interactions (Figure 1) are modeled with interaction constraints in the second (outer) optimization problem capturing the exchange of metabolites among different species and using maximization of overall community biomass as objective function (Zomorrodi and Maranas, 2012).

Bi-level optimization algorithms such as OptCom can capture metabolic interactions among members of a microbial community (Table 4). It is possible to incorporate ecological data of the community (i.e., species or guild presence/absence and abundance) as constraints in the second optimization problem. The observed growth rates of individual species can be used to define (constrain) the biomass flux of internal guild models. Food chains, substrate competition, syntrophy, and product inhibition can be modeled using bi-level optimization approaches. For instance, OptCom can be used for assessing the optimal growth rate for different members in a microbial community and subsequently making predictions regarding metabolic exchange given the identified optimal levels (Zomorrodi and Maranas, 2012). An advantage of the bi-level optimization approach is that it can also be coupled with differential equations to generate dynamic models (Zomorrodi et al., 2014). Theoretically, it is possible to include metabolic and species abundance data for an indefinite number of species or guilds. However, this remains challenging given the gaps in knowledge of species identities and metabolic details of complex communities. The bi-level simulation approach has the disadvantage of requiring two rounds of data processing and simulation, which can be computationally burdensome. In addition, using two types of data processing introduces some rounding error (Taffs et al., 2009). Finally, manual selection of ecologically interesting modes from individual models requires a priori knowledge and can significantly influence the solution.

### Dynamic-SMN (hybrid)

This approach couples the rate predictions of SMN models with differential equations that capture the dynamic response of the biological process with respect to compounds concentration, temperature, or pH. The main attribute of hybrid models is that they can predict reaction rates together with compound concentrations across a time period, which is a major advantage in applications such as process optimization. Consequently, interactions that depend on changes in substrate concentration or species abundance can be modeled with this approach. Dynamic-SMNs have been applied to model single species (Mahadevan et al., 2002; Hjersted et al., 2005; Çalik et al., 2011) as well as multiple species (Scheibe et al., 2009; Zhuang et al., 2011). This hybrid approach has been previously referred to as dynamic FBA (dFBA) (Mahadevan et al., 2002). Here, we refer to this method as dynamic-SMN rather than dFBA to avoid the assumption that the intracellular flux distribution can only be obtained via FBA and not other simulation algorithms such as flux variability analysis or random sampling.

Dynamic-SMN models are formed by three types of equations: (i) kinetic and (ii) differential equations, both capturing the process dynamics; and (iii) stoichiometric reaction equations of each modeled species/guild (*S*^*k*^), which capture the biochemical transformations (Figure 6). Initial conditions of the simulated system must be defined a priory (i.e., initial concentration of substrates (*X*_*i*_) and species biomass ($X_{biomass}^{k}$)). Also the analyzed time period is subdivided into discrete time intervals so that simulation length and the number of intervals must be defined (e.g., a simulation of 2 h using 7200 time intervals of 1 s each). During the simulation, the following four subroutines are executed for each time interval: (i) the substrates and biomass concentrations are used in the kinetic equations to estimate the uptake rate of substrates ($v_{Substrate\;uptake}^{k}$) for each species *k*. A kinetic equation (e.g., Monod or Logistic) is used for each substrate of interest related to each modeled species. For instance, two Monod equations are included in a model that capture the consumption of one substrate by two species; (ii) the obtained $v_{Substrate\;uptake}^{k}$ values are used as constraints of the respective *S*^*k*^ to solve the optimization problem (e.g., via FBA); (iii) the predicted production rates for compounds of interest and biomass ($v_{i}^{k}$ and $v_{biomass}^{k}$) are used in differential equations to obtain the change of concentrations Δ*X*_*i*_ and $\Delta X_{biomass}^{k}$; (iv) new concentrations at the end of the simulation step ($X_{i}^{NEW}$ and $X_{biomass}^{k\;NEW}$) are calculated by adding $\Delta X_{biomass}^{k}$ and Δ*X*_*i*_ to the initial concentrations *X*_*i*_ and $X_{biomass}^{k}$. The new concentrations are used as a starting point for the next itineration of subroutines executed for the next time interval. This continues until the simulation length is reached. At each time interval, the flux constraints for each organism vary based on the substrate concentration at that particular time, leading to dynamic variations. The underlying assumption of this approach is that the speed of processes inside the cell are faster than the changes in the surrounding environment, which allows the kinetics of environmental factors to be defined (e.g., substrate concentration change), without defining intracellular kinetic processes (Mahadevan et al., 2002).

Dynamic-SMN models captures both metabolic complexity and metabolic dynamism. The approach is particularly well suited to model microbial interactions in heterogeneous environments (e.g., batch cultures), as it does not assume constant yield coefficients (Schuetz et al., 2007). Because the majority of environmental bioprocesses (e.g., wastewater biotreatment and soil bioremediation) display time dependent dynamics, this approach has the potential to truly capture their behavior (Table 4). For instance, dynamic-SMN has been applied successfully to study bioremediation processes with mixed microbial populations (Zhuang et al., 2011; Embree et al., 2015). Scheibe et al. (2009) coupled a genome-scale SMN model of *Geobacter sulfurreducens* to a soil reactive transport model (HYDROGEOCHEM) to define *in situ* bioremediation strategies for uranium spills in soil. By representing an aquifer as a numeric grid, the hybrid model simulates time and space dependent hydrological, geochemical, and metabolic processes in the spill area. Another innovative tool based on SMN models is the Computation of Microbial Ecosystems in Time and Space (COMETS) (Harcombe et al., 2014). The approach couples dynamic-FBA simulations with extracellular-compounds diffusion models, which makes it possible to track not only the temporal but also spatial dynamics of multiple microbial species in complex environments with a complete genome scale resolution (Zomorrodi and Segrè, 2015). The COMETS approach has been applied successfully to identify the spatial arrangements of different species colonies in engineered microbial communities. Another novel tool is the Microbial Community Modeller (MCM) which combines genome-based model construction with statistical analysis and calibration to experimental data in a single platform (Louca and Doebeli, 2015). MCM has been used to simulate successional dynamics in single-species evolution experiments, and pathway activation patterns observed in microarray transcript profiles (Louca and Doebeli, 2015).

As a drawback, this approach is computationally demanding as several SMN simulations have to be performed to analyze the entire time period. Additionally, model calibration can be tedious because of the need to adjust many kinetic parameters including maximum reaction rates *v*_*max*_ and affinity constants *K*_*m*_ of kinetic equations (Makinia, 2010). Also, the maximum number of microbial and metabolic species depends on the computational hardware capacity. Nevertheless, given the complexity of defining kinetic equations for each modeled guild, this approach is best suited to simulated interactions within small communities of two to five guilds.

### Comparing approaches

Advantages and disadvantages of the approaches can be compared in terms of their required input data, their generated output data and their implementation (Table 5). In terms of input data, all the approaches except the lumped approach require extensive information about metabolic reactions and pathways from different species/guilds. Species presence/absence and abundance data can be used as model input for all approaches except for the lumped approach because the community is analyzed as a whole and the metabolism of individual species is not captured. Metabolic information from systems with a large number of species or guilds is better captured using lumped and bi-level optimization approaches because each species is modeled independently. Nevertheless, the computational requirement to run bi-level optimization simulations with multiple SMN can be significant. Presence/absence data of functional genes, enzymes or metabolites can be captured using all the approaches by defining gene-protein-reaction associations with Boolean rules (Thiele and Palsson, 2010; Lewis et al., 2012). Different approaches yield different output data and information. In general all the approaches are appropriate to obtain metabolic fluxes at the community level (i.e., overall production and consumption rates of compounds) but the dynamic-SMN additionally provides compound concentration profiles across time. Quantification of physiological attributes (fluxes) at the species/guild level (i.e., intracellular fluxes) can be generated with all the approaches except with lumped models. Similarly, microbial interactions among community species and guilds (i.e., exchange and competition of metabolites) can be quantified using all the approaches except the lumped approach as this does not contemplate the exchange of compounds between species. The temporal and spatial dynamics of compound concentrations can only been captured with the dynamic-SMN approach. In contrast, the lumped, multi-compartment and bi-level optimization approaches provide rates of metabolic reactions at a specific steady state.

**Table 5:** **SMN approaches to model microbial communities and their capabilities**.

	**Model capability**	**Lumped**	**Compartment per guild**	**Bi-level optimization simulation**	**Dynamic-SMN**
Required Input data	Requires extensive metabolic pathway information from different species	Not required	Required	Required	Required
	Captures species presence/absence	Limited	Optimal	Appropriate	Optimal
	Captures species abundance	Limited	Appropriate	Appropriate	Optimal
	Captures information from large number of species/guilds	Appropriate	Limited	Appropriate	Limited
	Captures functional gene, enzyme or metabolite presence/absence	Appropriate	Appropriate	Appropriate	Appropriate
	Captures gene-protein-reaction association	Appropriate	Appropriate	Appropriate	Appropriate
Generated output data	Quantifies physiology at community level	Appropriate	Appropriate	Appropriate	Optimal
	Quantifies physiology at species/guild level	Limited	Appropriate	Appropriate	Appropriate
	Quantifies inter species interactions	Limited	Appropriate	Optimal	Optimal
	Describes temporal and spatial changes of compound concentration (capturing process dynamic)	Possible	Possible	Limited	Optimal
Implementation	Easy to develop (network reconstruction)	Easy	Moderate	Challenging	Challenging
	Easy to calibrate	Easy	Moderate	Moderate	Challenging
	Computationally demanding	Low	Low	High	High
	Optimal environmental system to be described	Natural and engineered systems without well-defined species populations	Natural and engineered systems with low species richness	Natural and engineered systems with high species richness	Engineered systems with low species richness

The best approach for use depends on the system under analysis (Table 5). Natural and engineered systems without well-defined species populations are best modeled with lumped models. The lumped approach represents the coarsest methodology, requires the least *a priori* information and is easier to implement than alternative approaches. It can be used when other approaches cannot (due to complexity) or should not (due to lack of detailed data). These advantages are balanced against its tendency to overestimate the metabolic potential. This is unsurprising, as real communities are not super-organisms. Individuals are membrane-separated and must contend with the logistics associated with matter and energy transport. Consequently, the lumped technique is best for initial work on “poorly” characterized systems (Taffs et al., 2009). Natural and engineered systems with low species richness are best characterized using the compartment per guild approach. The compartmentalized community analysis method has the advantage of intuitive tractability and separates activity and function by guild, but requires substantially more knowledge of the community than the pooled reactions approach. The compartmentalized method also lends itself uniquely to investigation of the robustness of specific consortium interaction types (Taffs et al., 2009). Natural and engineered systems with high species richness are best characterized using the bi-level optimization approach. This approach has properties very similar to the compartment per guild approach, but with the important advantage of easy scalability, achieved by solving each species' SMN separately. The approach also provides additional ecological insight into the competitive strategies underlying each guilds function. The bi-level simulation approach also easily captures interactions between different guilds as well as between members of the same guild expressing different physiologies. Finally, engineered systems with low species/guild richness are best analyzed using dynamic-SMN models as this approach is the only one that estimates concentrations of compound across temporal and spatial gradients.

## Applications of SMN modeling of microbial interactions

As more metabolic models of different organisms become available, the modeling of microbial communities becomes more feasible and relevant. SMN modeling has multiple applications for the analysis of microbial interactions and environmental bioprocesses (Table 4 and Figure 3), which are described in the following sections.

### Inference of metabolic mechanisms from observed data

In this application, experimental data is acquired and used as model input to generate estimations of intracellular metabolic rates and inter-species compound exchange rates. Experimental observations of community metabolism are then contrasted and interpreted under the light of model predictions (Pramanik et al., 1999; Chaganti et al., 2011). Because the SMN model includes detailed information of metabolic pathways, a mechanistic interpretation of the results obtained from experiments is possible (Rodríguez et al., 2006). In addition, SMN modeling can be used to infer ecological relationships in complex microbial communities, especially with regard to mechanisms of mass and energy transfer between guilds, and the relationship between species presence and its function in the community (Stolyar et al., 2007; Taffs et al., 2009). For example, in the Stolyar et al. (2007) study simulations helped reveal and clarify essential substrate assimilatory pathways and reaction stoichiometry by comparing simulation results with growth rates of experimental data.

### Process optimization

SMN models can be used to predict the likely outcome of new operation and management strategies for experiments or environmental processes (Scheibe et al., 2009). It is recognized that the investigation of the optimal process operation can be most effectively performed by adopting a model-based methodology (Dias et al., 2005). The model is used to develop experimental designs and hypotheses about relevant metabolic pathway or points of metabolic regulation and modulation (Pramanik et al., 1999). Intracellular flux distributions for different environmental scenarios can be calculated and culture feeding scenarios can be optimized with simulations targeting maximal compound productivity and/or desired composition (Dias et al., 2008). This is particularly useful for linking specific operational parameters to bioprocess product formation. For instance, Dias et al. (2005) and Pardelha et al. (2012) developed a process model based on SMN modeling to optimize the PHB productivity by mixed cultures. These studies aimed to explore optimal carbon sources and ammonia-feeding strategies that maximize both the final intracellular PHB content as well as the volumetric productivity (Dias et al., 2005). Computational tools such as the Search for Interaction-Inducing Media (SIM) algorithm identifies the set of media that support the growth of multi-species cultures and predicts the class of interaction they induce (Klitgord and Segrè, 2010). In summary, the inclusion of genome information in SMN models can be used to select optimal combinations of microbial taxa or genes to promote more efficient substrate degradation and/or production.

### Analysis of high-throughput “omic” data

Metabolic network models have successfully helped in the interpretation of transcriptomic, proteomic and metabolomic data from single-species cultures. As mentioned before there is a plethora of published computational methods to analyze SMN models (Durot et al., 2009; Kim et al., 2012; Lewis et al., 2012) including omic data mining. For instance, proteomic and transcriptomic data have been successfully used as constraints of SMN models and interpreted through the Parsimonious FBA (pFBA) (Lewis et al., 2010) and the ME-modeling framework (Lerman et al., 2012) among others. In a similar way, metabolomics data can be interpreted in the light of SMN models using Network Embedded Thermodynamic (NET; Kümmel et al., 2006), network topology (Çakir et al., 2006), or shadow price (Reznik et al., 2013) analyses. Depending on the intended application, proper method selection is important as results from the same model can differ significantly depending on the method used. For example, Machado and Herrgård (2014) systematically evaluated eight SMN methods for analysis of transcriptomic data and concluded that none of the methods outperform the other for all the tested cases. Recent advances in the use of high-throughput sequencing and whole-community analysis techniques, such as meta-genomics and meta-transcriptomics, are making genomic information available from microbial communities. However, due to the complexity or low reliability of the information generated in many studies, “meta-omic” data may remain without meaning or usefulness. In principle, SMN models can be used to analyze and interpret “meta-omics” data by extending the computational methods developed for “omic” data analysis. However, as best of authors knowledge, SMN models have only been used for metagenomics and other “meta-omics” data analysis in very few studies (i.e., Pérez-Pantoja et al., 2008; Nagarajan et al., 2013; Embree et al., 2015). This gap in the research field opens a promising research opportunities for groups specialized in developing systems biology and bioinformatics tools.

### Design of novel catalytic pathways and microbial associations

The most innovative application of metabolic network modeling of mixed-microbial communities is to discover and design novel microbial associations and catalytic pathways. The real power of computational biology techniques relies on their ability to rapidly test thousands of metabolic variations or combinations without developing wet experiments or generating mutants. For instance, it is possible to computationally generate artificial microbial ecosystems without re-engineering microbes themselves, but rather by predicting their growth on appropriately designed media. This approach is of particular relevance to environmental biotechnology, given the restrictions on the use of genetically modified organisms in bioremediation strategies. SMN models can be used to identify novel environmental conditions to co-cultivate two or more species by inducing mutualistic or commensal interaction interactions (Stolyar et al., 2007; Klitgord and Segrè, 2010). For example, in the study done by Klitgord and Segrè (2010) 21 models were generated using paired combinations of seven SMN models of different species. From the simulations of these paired models, several putative growth media formulations were identified to induce novel commensal or mutualistic relationships between the species. Naturally, further experimentation is required to confirm model's predictions, but these experiments would be based on a robust hypothesis generated *a priori*. In another relevant *in silico* study by Taffs et al. (2009), three SMN models were used to map the novel pathways generated by the metabolic networks of three species connected to each other via the exchange of substrate and products.

SMN models can help to explore community enzymatic potential to assemble novel interspecies catalytic pathways. Novel pathways can be formed by inducing interactions between different organisms rather than—or in addition to—genetically modified organisms (Chiu et al., 2014). This is beneficial as firstly one could use the metabolic potential of organisms that may be hard to genetically manipulate. Secondly, communities may have a more robust metabolic performance than individual modified species, in which specific mutations can revert the genetic modification. In this sense, symbiotic interactions, e.g., to biodegrade a pollutant, may arise more readily through environmental fluctuations than genetic modifications (Klitgord and Segrè, 2010; Zomorrodi and Segrè, 2015). Using a multi-compartment approach, Klitgord and Segrè (2010) developed the Search for Exchanged Metabolites (SEM) algorithm to verify potential interactions between a pair of organisms by generating lists of metabolites able to be exchanged by a defined pair of species/guilds. This approach has huge potential for discovering and designing novel microbial interactions.

## Challenges of modeling microbial communities using metabolic networks

Whether developed for individual species or microbial communities, SMN models have inherent and important challenges that must be considered, including: (i) valid metabolic networks are difficult to develop as details of many metabolic reactions and pathways are unknown, this is especially true for secondary metabolic pathways (Durot et al., 2009; Thiele and Palsson, 2010; Kim et al., 2012); (ii) model outputs (e.g., intracellular flux estimations) can be uncertain as model predictions do not necessarily reflect real fluxes, also models can provide multiple solutions to a single problem. Given these features, extensive model curation and calibration against experimental data is required (Varma and Palsson, 1995; Edwards et al., 2001; Kumar and Maranas, 2009; Perez-Garcia et al., 2014a); (iii) in principle SMN models and their analysis methods simulate cellular systems at steady state and do not consider the accumulation of metabolic compounds, which limits their application to study dynamic systems (Savinell and Palsson, 1992; Varma and Palsson, 1994a); (iv) generally SMN models and their analysis methods have to employ artificial assumptions that can bias the model outputs (i.e., selection of artificial objective functions for model solving) (Segrè et al., 2002; Schuetz et al., 2007; Feist and Palsson, 2010); and (v) in principle SMN models and their analysis methods disregard gene regulatory processes, gene expression profiles, and do not consider enzyme accumulation and kinetics (Pramanik and Keasling, 1997; Price et al., 2003). On top of these challenges, modeling of microbial interactions and communities adds significant layers of complexity which are described below.

The starting point for SMN models is the information on species genome and gene functions. However, it is important to keep in mind that genome annotations in databases may have errors, and that identifying genes that encode for catalytic enzymes it is not always straight forward. Furthermore, any genome will contain a good portion of genes of unknown function, and large parts of the genome encode proteins involved in non-metabolic processes (Zengler and Palsson, 2012). Knowledge of the most important microbial guilds involved in the performance of a given mixed microbial culture is a prerequisite. Once the functional guild or species is identified, whole-genome sequences in conjunction with detailed physiological experiments enable SMN models to be generated. De novo genome annotation is a challenge by itself.

Also, determining the abundance of individual microbial species/guilds in the system of interest is essential to develop more realistic SMN models for microbial communities. This is because metabolic fluxes for each species can be scaled to the amount of species abundance, which makes it possible to evaluate the contribution of each species to the whole community performance (Khandelwal et al., 2013; Perez-Garcia et al., 2016). Biological abundance can be quantified directly with techniques like fluorescent *in situ* hybridization (FISH), quantitative polymerase chain reaction data (qPCR), reverse transcription qPCR (RT-qPCR) and flow cytometry (Wagner and Loy, 2002; Daims et al., 2006). Cultivation-independent approaches, such as metagenomics, metatranscriptomics, and metaproteomics, target the community as a whole and can also provide insights into species/guild abundance, but they have limited resolution at the species or strain level (Zengler and Palsson, 2012). An outstanding question is whether SMN models can be applied to the much larger number of interacting species present in most ecosystems, and whether large modular stoichiometric models are going to be useful and necessary.

SMN modeling efforts of microbial communities should also focus on identifying suitable objective functions. The solution space of a given model is not entirely a feature of network structure, but also a function of the chosen objective function (*Z*) and constraints (α_*j*_ ≤ *v*_*j*_ ≤ β_*j*_). SMN modeling uses optimization principles to estimate reaction rates of a given metabolic network. As mentioned before, generally maximization of biomass production per unit of substrate is the most suitable objective function for single-species metabolic networks (Segrè et al., 2002; Schuetz et al., 2007; Feist and Palsson, 2010). This seems to also hold true for multi-species metabolic networks (Klitgord and Segrè, 2010; Perez-Garcia et al., 2016). However, it is important to acknowledge that no single objective can be used to predict experimental data under all conditions of a given biological system. Thus, it is necessary to identify the most relevant objective for each condition. To do this, formal studies investigating the use of different objective functions to model a given microbial community under different environmental conditions are required. Under nutrient scarcity, cell metabolism normally supports efficient biomass formation with respect to the limiting nutrient. This operational state appears to have evolved under the objective to maximize either the ATP or biomass yield (synonymous to the frequently used maximization of growth rate objective). For cultures under conditions that allow unlimited growth, in contrast, energy production is clearly not optimized *per se* because cells secrete or accumulate large amounts of organic compounds, instead of using them for energy generation (Schuetz et al., 2007). Investigations of different objective functions on models' predictive capabilities will enhance the reliability and robustness of SMN models of microbial interactions.

An appropriate and rigorous model calibration is required to achieve a high level of confidence that the estimated rates of metabolic reactions are a valid representation of the metabolic activity of real cells. Surprisingly there is no many methods available to compare experimental versus predicted data. Although SMN model calibration involves: (a) an accurate definition of stoichiometric equations based strictly on proven biochemical data by which the chemical compounds and capabilities of mass transformations of the system are defined; (b) correct definition of objective function(s) and the solving method of the optimization problem; and (c) obtaining high fitness (e.g., high correlation) between experimental data and model predictions—the model must mimic at least the consumption and production rates of compounds observed in cultures. Most estimated rates of metabolic reactions cannot be measured experimentally and therefore cannot be validated directly. Nonetheless, data from microarray transcript profiles, transcriptomics, proteomics, metabolomics, and fluxomics [13C-based metabolic flux analysis (13C-MFA)] methods can be used to reduce uncertainty and increase the accuracy of the model‘s predictions (Figure 3).

Finally, it is important to recognize the different dynamics scales of biological system. At least four scales can be pointed: evolutionary, environmental, population and cell regulatory. For instance it is suggested that while community's organism lineages fluctuate extensively through time and conditions, the functional content of microbial communities displays stability and correlations with environmental parameters (Klitgord and Segrè, 2011). As asked by Klitgord and Segrè (2011): “Will the often large fluctuations in population dynamics dwarf the importance of regulatory dynamics within individual species? How can one model and understand the interplay between these two types of dynamic phenomena and their role in shaping microbial ecosystems?” Answering these important questions can significantly impact both, basic biological and evolutionary concepts as well as practical community culturing applications. None of our current computational tools can simultaneously capture the dynamics in all the mentioned scales. However, modeling approaches such as COMETS, HYDROGEOCHEM, OptCom, and MCM are pioneering tools to integrate different scale dynamics.

## Conclusions

SMN modeling and systems biology can contribute to a comprehensive understanding of microorganisms, their interaction with other species in a community and their interplay with their environment. Understanding how interaction among cells enables the spread of information and leads to dynamic population behaviors is a fundamental problem in biology (Xavier, 2011). Meaningful insight into the interaction of microorganisms with other organisms and the environment has often been hampered by the fact that microbial communities are extremely complex (Zengler and Palsson, 2012). Nowadays, realistic microbial community models are long way off, because our knowledge of microbial interactions is still incomplete. Even if all this knowledge were available, microbial community modeling faces daunting challenges as microbiomes are highly complex, nonlinear, evolving systems that can be chaotic and therefore unpredictable (Faust and Raes, 2012). SMN modeling approaches can help to address such complexity because most interactions between different microorganisms influence their metabolism. Identification of the most important microbial guilds involved in the analyzed environmental system is a prerequisite to characterizing it using an SMN model. Once the functional guild or species is identified, whole-genome sequences, in conjunction with detailed physiological experiments, enable SMN models to be generated for the identified organisms. Species-specific SMN models are then used to build up community models using one of the four modeling approaches. Modeling of microbial communities requires the description of molecular mechanisms that describe species interactions, such as competition, food chains, and inhibition (Figure 1). Nevertheless, it is important to recognize the SMN models do not capture strict ecological interaction (e.g., predation, parasitism) but rather metabolic interactions that can have ecological repercussions within the community (Figure 1).

The successful application of SMN modeling to characterize microbial interactions in natural and engineered environmental systems requires recognizing and modeling several abiotic factors influencing process performance (Figure 2). The chemical factors of the process include carbon source availability, nutrient availability, electron donor/acceptor availability, pH, and chemical stressors. The physical factors are those imposed by the micro/macrogeography of the organisms location and include, for example, humidity, conductivity, temperature, pressure, diffusion, and texture and density of the extracellular matrix (de Lorenzo, 2008). As this complexity increases, there is a need to develop a new set of fundamental principles, concepts and algorithms that will further reveal the secrets of microbial and cellular communities (Zengler and Palsson, 2012). SMN modeling of microbial communities and subsequent computer simulations are tools that can lead to a better understanding of the microbial cell and will undoubtedly contribute significantly to the field of environmental biotechnology. Microbial ecology and environmental biotechnology are inherently tied to each other. The concepts and tools of microbial ecology are the basis for managing processes in environmental biotechnology; and these processes provide interesting ecosystems to advance the concepts and tools of microbial ecology (Rittmann, 2006). Revolutionary advancements in molecular tools to understand the structure and function of microbial communities are strengthening the power of microbial ecology. A push from advances in modern materials along with a pull from a societal need to become more sustainable is enabling environmental biotechnology to create novel processes (Rittmann, 2006).

Systems biology tools such as SMN modeling have created the opportunity to develop the next generation of models of environmental process involving biological transformations. Nowadays, cheaper molecular biology and genomics, proteomics, and metabolomic techniques allow us to identify and quantify specific microbial species, full genome sequences, gene expression activity and metabolic compounds (Lovley, 2003). In addition, metabolomics can be applied to elucidate the biodegradation pathways of pollutants by identifying and quantifying dozens or even hundreds of compounds in a single sample (Villas-Bôas and Bruheim, 2007). Powerful computers are becoming cheaper, and new computation algorithms for data mining and model simulation are generated more readily (Lewis et al., 2012). The significant advantage of SMN models in this context is that they can incorporate the data generated with these new techniques and tools to produce a more accurate and realistic quantification of microbial processes. Therefore advanced metabolic models like these can serve as a bridge between molecular/biochemical research and environmental engineering practices, effectively functioning as a tool that can better link the work of microbiologists and engineers (Oehmen et al., 2010). SMN models are tools with potential to be used not only in research but also in applications such as biogeochemical cycle analysis and techno-economics, through linkages with hydrodynamic and geochemical models. The methods and applications detailed in this review and future developments in this area will help to decipher patterns of compounds and energy flow in environmental systems; these capabilities must be employed for the sustainable and integral development of human socio-economic activities within nature.

## Author contributions

OP: Main literature revision and initial manuscript preparation. GL: Revision of sections focusing in microbial ecology and interactions and editorial improvement. NS: Revision of sections focusing in mathematical modeling and main paper editor.

### Conflict of interest statement

The authors declare that the research was conducted in the absence of any commercial or financial relationships that could be construed as a potential conflict of interest.
